# Translational upregulation of *Aurora-A* by hnRNP Q1 contributes to cell proliferation and tumorigenesis in colorectal cancer

**DOI:** 10.1038/cddis.2016.479

**Published:** 2017-01-12

**Authors:** Chien-Hsien Lai, Yu-Chuan Huang, Jenq-Chang Lee, Joseph Ta-Chien Tseng, Kung-Chao Chang, Yen-Ju Chen, Nai-Jhu Ding, Pao-Hsuan Huang, Wen-Chang Chang, Bo-Wen Lin, Ruo-Yu Chen, Yu-Chu Wang, Yi-Chien Lai, Liang-Yi Hung

**Affiliations:** 1 Institute of Bioinformatics and Biosignal Transduction, College of Bioscience and Biotechnology, National Cheng Kung University, Tainan 70101, Taiwan; 2 Department of Surgery, College of Medicine, National Cheng Kung University Hospital, Tainan 70403, Taiwan; 3 Department of Pathology, College of Medicine, National Cheng Kung University Hospital, Tainan 70403, Taiwan; 4 Graduate Institute of Medical Science, College of Medicine, Taipei Medical University, Taipei 11031, Taiwan; 5 Department of Biotechnology and Bioindustry Sciences, College of Bioscience and Biotechnology, National Cheng Kung University, Tainan 70101, Taiwan; 6 Institute for Cancer Biology and Drug Discovery, College of Medical Science and Technology, Taipei Medical University, Taipei 11031, Taiwan

## Abstract

By using RNA-immunoprecipitation assay following next-generation sequencing, a group of cell cycle-related genes targeted by hnRNP Q1 were identified, including Aurora-A kinase. Overexpressed hnRNP Q1 can upregulate Aurora-A protein, but not alter the mRNA level, through enhancing the translational efficiency of *Aurora-A* mRNA, either in a cap-dependent or -independent manner, by interacting with the 5′-UTR of *Aurora-A* mRNA through its RNA-binding domains (RBDs) 2 and 3. By ribosomal profiling assay further confirmed the translational regulation of *Aurora-A* mRNA by hnRNP Q1. Overexpression of hnRNP Q1 promotes cell proliferation and tumor growth. HnRNP Q1/ΔRBD23-truncated mutant, which loses the binding ability and translational regulation of *Aurora-A* mRNA, has no effect on promoting tumor growth. The expression level of hnRNP Q1 is positively correlated with Aurora-A in colorectal cancer. Taken together, our data indicate that hnRNP Q1 is a novel *trans*-acting factor that binds to *Aurora-A* mRNA 5′-UTRs and regulates its translation, which increases cell proliferation and contributes to tumorigenesis in colorectal cancer.

Heterogeneous nuclear ribonucleoproteins (hnRNPs) are a large group of RNA-binding proteins that associate with the heterogeneous nuclear RNAs (hnRNAs) transcribed by RNA polymerase II in eukaryotic cells. All members of the hnRNP family share a similar protein structure, consisting of at least one RNA-binding domain (also called the RNA recognition motif, RRM) and combine with other auxiliary domains such as the RGG box or the acidic domain responsible for protein–protein interactions or additional RNA-binding abilities.^1^ HnRNP Q (also called SYNCRIP or NSAP1) is an AU-rich RNA-binding protein and shares approximately 80% sequence identity with hnRNP R.^2, 3, 4^ In humans, seven hnRNP Q isoforms, which result from alternative splicing of *SYNCRIP*, have been identified. HnRNP Q members consist of one acidic domain (AcD) at the N-terminus that is involved in protein–protein interaction, three RNA-binding domains (RBDs) in the central region and one RGG box at the C-terminus for binding to RNA. HnRNP Q proteins have multifunction in regulating mRNAs, including pre-mRNA splicing,^4, 5^ mRNA editing,^6, 7^ transport,^8, 9^ turnover^10, 11^ and primarily regulating translation.^12, 13, 14^ Among the hnRNP Q proteins, hnRNP Q1 (isoform 6, NP_001153149.1) is the smallest isoform, ubiquitous and the most abundant member of the family. HnRNP Q1 lacks the second nuclear localization signals (NLS) in the C-terminus and has a subcellular localization in both the cytoplasm and nucleus, differing from the other hnRNP Q isoforms which are limited to the nucleus.^3, 5^ The cytoplasmic localization of hnRNP Q1 may respond to most of the mRNA metabolic processes occurring in the cytoplasm, including mRNA transport, translation and turnover, all of which can directly affect the protein production of target mRNAs.

The internal ribosome entry site (IRES) is a specific secondary structure located at the 5′-UTR of mRNA. The IRES can recruit a ribosome to initiate translation without the formation of 5′-cap recognized complex. During mitosis, cap-dependent translation is suppressed, and IRES-mediated translation is the alternative way for protein synthesis. According to the literatures, hnRNP Q can act as a positive regulator of the IRES.^12, 15^ However, some reports indicate that hnRNP Q can target the 3′-UTR of mRNAs and then disrupt poly(A)-binding protein (PABP)-mediated circular mRNA formation to repress cap-dependent translation.^14, 16, 17^ Therefore, the role of hnRNP Q in regulating translation might depend on the binding sites of mRNAs. A previous report indicated that hnRNP Q is involved in the maintenance of cell proliferation in colon cancer cells by associating with galectin-3, but the underlying mechanism is still unclear.^18^ Furthermore, the specific target genes regulated by hnRNP Q have not been completely identified.

Our previous report showed that the translation of *Aurora-A* mRNA can be upregulated in colorectal cancer by membrane receptor-mediated downstream signaling, and the 5′-UTR of *Aurora-A* mRNA is critical for translational regulation.^19^ In the present study, we found that hnRNP Q1 can promote cell proliferation and translationally regulates a group of cell cycle-related genes including *Aurora-A*. The expression of hnRNP Q1 is positively correlated with Aurora-A in human colorectal cancer tissues. Our results suggest that hnRNP Q1 is a novel *trans*-acting factor that binds to the *Aurora-A* 5′-UTR and regulates translation, both in cap-dependent and IRES-dependent manners, which may increase cell proliferation and contribute to the tumorigenesis of colorectal cancer.

## Results

### HnRNP Q1 enhances cell proliferation through regulating a group of cell cycle-related genes

**Figure 1:**
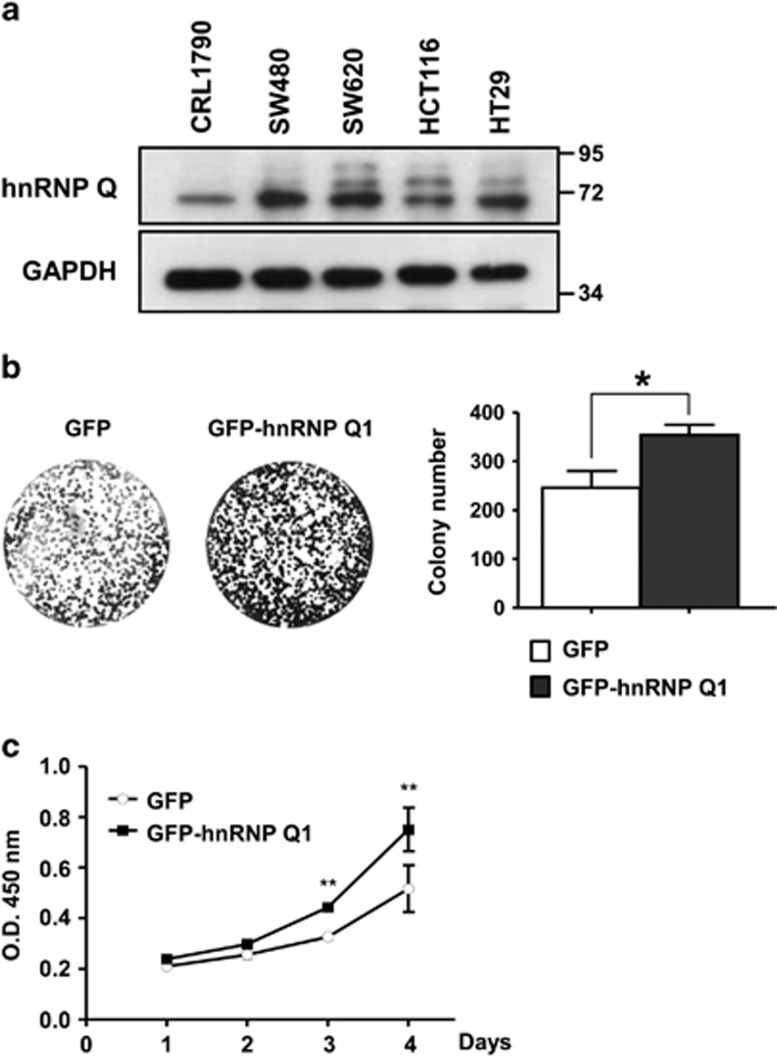
HnRNP Q1 is overexpressed and can enhance cell proliferation in colorectal cancer cells. (**a**) Total cell lysates from the normal colon cell line CRL1790 or from colorectal cancer cell lines SW480, SW620, HCT116 and HT29 were collected for western blot analysis by using anti-hnRNP Q antibody. GAPDH was used as a loading control. (**b**) SW480 cells with GFP-hnRNP Q1 stable expression exhibit increased colony-formation ability compared with cells with GFP expression only. Quantitative results of the colony-formation assay are also shown; *P<0.05. (**c**) The cell proliferation rate in SW480 cells with GFP or GFP-hnRNP Q1 expression was determined by using the CCK-8 kit; **P<0.01

To investigate the role of hnRNP Q1 in tumorigenesis, the expression of hnRNP Q1 in colorectal cancer cell lines was first examined. The results indicated that hnRNP Q1 is overexpressed in colorectal cancer cell lines than the normal colon cell line CRL1790 (Figure 1a). To further clarify the physiological function of hnRNP Q1, SW480 cells were permanently transfected with GFP-hnRNP Q1 or GFP (Supplementary Figure S1A), and then we performed a colony-formation assay. The data showed that cells with GFP-hnRNP Q1 increased their colony-formation abilities relative to cells with GFP (Figure 1b). The cell proliferation assay further showed a higher proliferation ability of GFP-hnRNP Q1-overexpressing cells than GFP-expressing cells (Figure 1c). These results suggest that hnRNP Q1 may increase the cell growth ability during tumor formation.

To further identify the hnRNP Q1-associated mRNAs in cancer cells, an RNA-immunoprecipitation (IP) assay followed by next-generation sequencing (NGS) was performed in GFP-hnRNP Q1-overexpressed cells. The subcellular localization of hnRNP Q1 was first characterized, and the results indicated that only hnRNP Q1 existed in both the nucleus and the cytoplasm, in contrast to the other hnRNP Q variants, hnRNP Q2 and Q3 (Supplementary Figure S1B). Supplementary Table S1 shows the top 10 most significant groups of hnRNP Q1-associated mRNAs, of which more than half of the hnRNP Q1-associated targets are involved in RNA metabolic processes, which agrees with previous reports on the biological functions of hnRNP Q1.^4, 8, 10^ Interestingly, a group of cell cycle- and mitosis-related genes are the potential targets of hnRNP Q1 (Supplementary Tables S1 and S2). This result suggests hnRNP Q1 may promote cell proliferation by regulating a group of cell cycle-related genes. Among these genes, we selected *Aurora kinase A* (*AURKA*) for the following study because of its roles in mitotic entry and tumorigenic capacity.^20^

### HnRNP Q1 directly binds to *Aurora-A* mRNA 5′-UTR

**Figure 2 fig2:**
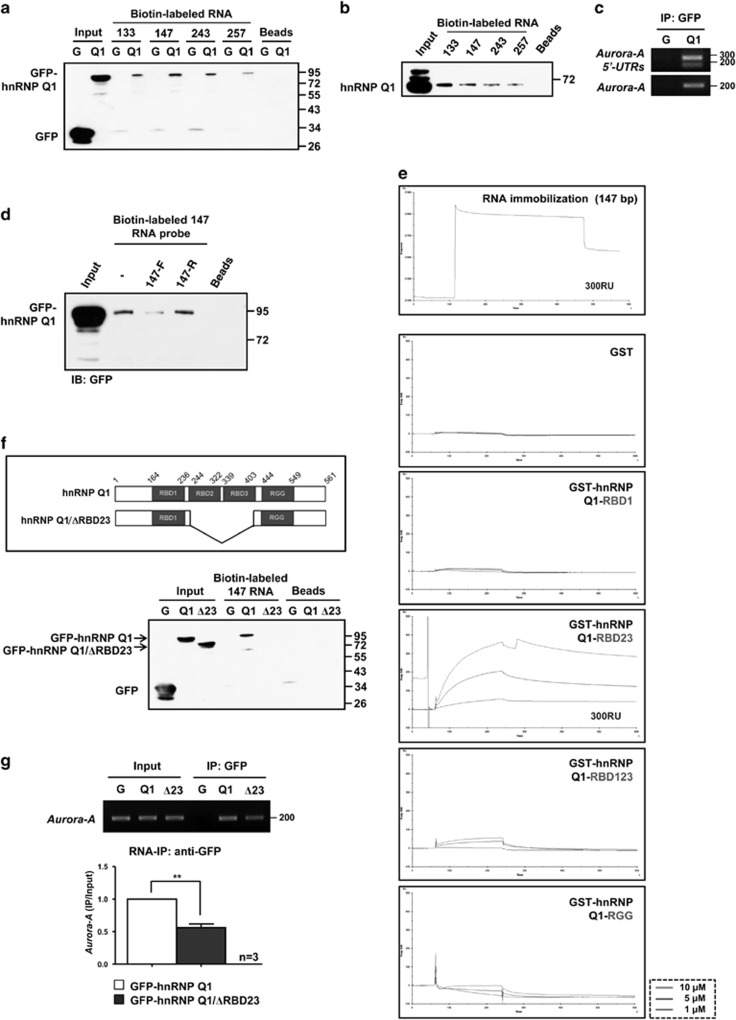
HnRNP Q1 directly binds to *Aurora-A* mRNA 5′-UTR through RBD23 domains. (**a** and **b**) Four types of biotin-labeled *Aurora-A* mRNA 5′-UTR RNA probes – 133 nt, 147 nt, 243 nt or 257 nt were incubated with total cell lysates from GFP (G) or GFP-hnRNP Q1 (Q1)-expressing cells (**a**) or SW480 cells (**b**). The pulled-down proteins were separated by SDS-PAGE and immunoblotted with anti-GFP or anti-hnRNP Q antibody. (**c**) Cytoplasmic lysate from GFP (G) or GFP-hnRNP Q1 (Q1)-expressing cells were collected for the RNA-immunoprecipitation (RNA-IP) assay using anti-GFP antibody. The precipitants were collected for RNA extraction and subjected to RT-PCR to assess the binding of *Aurora-A* mRNA or *Aurora-A* 5′-UTRs. (**d**) Biotin-labeled *Aurora-A* mRNA 5′-UTR 147 nt RNA probe was incubated with cell lysates from GFP-hnRNP Q1-expressing cells in the presence of a twofold amount of unlabeled *Aurora-A* mRNA 5′-UTR 147 nt forward RNA probe (147-F) or reversed 147 nt RNA probe (147-R) as described in **a**. (**e**) Biotin-labeled *Aurora-A* mRNA 5′-UTR RNA probe (147 nt) was immobilized on a streptavidin (SA) sensor chip, and then GST-hnRNP Q1 proteins were subjected to an *in vitro* binding assay by SPR. The interaction between the *Aurora-A* 5′-UTR and GST-hnRNP Q1 proteins was quantified by the change of resonance units (RU) on the chip. Three different concentrations of GST-hnRNP Q1 proteins, 1 *μ*M, 5 *μ*M and 10 *μ*M, were used. (**f**) (Upper) Scheme illustrates RBD23-truncated hnRNP Q1 (GFP-hnRNP Q1/ΔRBD23). (Lower) Total cell lysates from GFP (G), GFP-hnRNP Q1 (Q1) or GFP-hnRNP Q1/ΔRBD23 (Δ23)-expressing cells were collected for biotin pull-down assay as described in **a**[Fig fig2]. (**g**) RNA-IP assay to investigate the interaction between GFP (G), GFP-hnRNP Q1 (Q1) and GFP-hnRNP Q1/ΔRBD23 (Δ23) with *Aurora-A* mRNA was performed as described in **c**[Fig fig2]. The associated *Aurora-A* mRNA with hnRNP Q1 was detected by PCR (upper) and real-time qPCR (lower); **P<0.01

According the NCBI database, there are six *Aurora-A* mRNA isoforms that result from alternative splicing of the 5′-UTR (Supplementary Figure S2A), and four of them are dominantly expressed in colorectal cancer and cancer cell lines (Supplementary Figure S2B).^19^ In contrast, normal colon cell line, CRL1790, expresses very low levels of these four *Aurora-A* mRNA isoforms (Supplementary Figure S2C). *In vitro* biotin pull-down assay showed that all four biotin-labeled *Aurora-A* mRNA 5′-UTR RNA probes could be associated with GFP-hnRNP Q1 (Figure 2a) or the endogenous hnRNP Q1 (Figure 2b). The *Aurora-A* mRNA 5′-UTR *exon1* or *exon1* variant (133 or 147 nt) showed a stronger binding affinity with hnRNP Q1. The interaction between endogenous hnRNP Q1 and *Aurora-A* mRNA 5′-UTRs was confirmed by RNA-IP assay (Figure 2c), and biotin pull-down competition assay further confirmed the interaction between *Aurora-A* mRNA 5′-UTR and hnRNP Q1 (Figure 2d).

Next, surface plasmon resonance (SPR) binding analysis was performed to identify the *Aurora-A* mRNA 5′-UTR interacting domain of hnRNP Q1. Four GST-hnRNP Q1 truncated proteins were generated to incubate with the *Aurora-A* 5′-UTR 147 nt RNA probe (Supplementary Figure S2D). The result showed that the RNA-binding domains 2 plus 3 (RBD23) of hnRNP Q1 could strongly interact with *Aurora-A* mRNA 5′-UTR in a dose-dependent manner (Figure 2e). *In vitro* biotin pull-down assay and RNA-IP analysis further demonstrated that RBD23-truncated GFP-hnRNP Q1 (GFP-hnRNP Q1/ΔRBD23) has an impaired interaction ability with *Aurora-A* mRNA (Figures 2f and g). Interestingly, the subcellular localization of GFP-hnRNP Q1/ΔRBD23, which still contains the NLS domain,^4^ is restricted to the nucleus (Supplementary Figure S3). In addition, *in vitro* biotin pull-down assay demonstrated that the *cis*-interacting element of *Aurora-A* 5′-UTR is located in the 5′-end of the 147 nt (Supplementary Figure S4). These results suggest that hnRNP Q1 can directly bind to the *Aurora-A* mRNA 5′-UTR, which has a higher affinity with *exon1*, through the RBD2 and RBD3 domains.

### HnRNP Q1 increases the translational efficiency of *Aurora-A* mRNA

**Figure 3:**
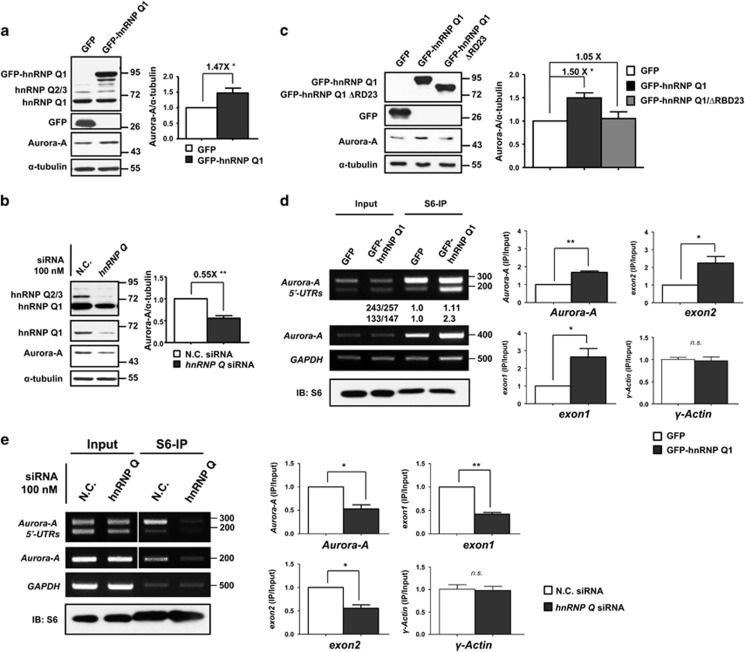
HnRNP Q1 translationally upregulates *Aurora-A* mRNA and increases Aurora-A protein expression. (**a** and **b**) SW480 cells transiently transfected with GFP, GFP-hnRNP Q1 (**a**) or control siRNA (NC), *hn RNP Q* siRNA (**b**) were collected to perform western blot analysis using antibodies as indicated. The quantitative results from three independent experiments of Aurora-A proteins expression level by western blot analysis are also shown; *P<0.05, and **P<0.01. (**c**) SW480 cells transiently transfected with GFP, GFP-hnRNP Q1 or GFP-hnRNP Q1/ΔRBD23 were collected for western blot analysis. The quantification of Aurora-A proteins expression from three independent experiments is shown; *P<0.05. (**d**) GFP or GFP-hnRNP Q1 expressed SW480 cells were collected for ribosomal protein S6-IP assay. The amount of *Aurora-A* mRNA or *Aurora-A* 5′-UTR isoforms in ribosomal complex was determined by RT-PCR (left) or RT-qPCR (right) from three independent experiments. The relative amount of *exon1*- (133/147) and *exon2*-containing (243/257) 5′-UTR of *Aurora-A* mRNAs immunoprecipitated by S6 protein were quantified by Scion Image as shown below. Upper: 243/257. Lower: 133/147. (**e**) SW480 cells were transfected with *hnRNP Q* siRNA for 48 h and then an S6-IP assay was performed as described above. The results of RT-PCR (left) and RT-qPCR (right) from three independent experiments are shown. Equal amount of immunoprecipitated S6 protein was verified by western blot. *γ-Actin* was used as negative control. *P<0.05, and **P<0.01; n.s., no significance

To clarify whether hnRNP Q1 is involved in the translational regulation of *Aurora-A* mRNA, the protein expression level of Aurora-A in GFP-hnRNP Q1-overexpressing or hnRNP Q1 knocked-down cells was investigated. The result showed that Aurora-A is increased in GFP-hnRNP Q1-expressing cells but decreased in *hnRNP Q* siRNA-transfected cells (Figures 3a and b). Neither the protein stability of Aurora-A (Supplementary Figure S5A) nor the expression level of *Aurora-A* mRNA, including all the four 5′-UTR variants, is altered in cells with different expression levels of hnRNP Q1 (Supplementary Figures S5B and C). Furthermore, GFP-hnRNP Q1/ΔRBD23 had no effect on Aurora-A protein expression (Figure 3c and Supplementary Figure S6A). *In vivo* translation assay showed that GFP-hnRNP Q1 could increase the translational efficiency of *Aurora-A* mRNA 5′-UTR *exon1* (133 nt) or *exon1* variant (147 nt) and weakly increase the translation of *exon2*-containing variants (243 and 257 nt; Supplementary Figure S6B). GFP-hnRNP Q1 has no effect on pGL3 promoter vector containing its own 5′-UTR and *WWOX*-5′-UTR-pGL3 containing the 5′-UTR of *WWOX* (Supplementary Figure S6C). In addition, GFP-hnRNP Q1/ΔRBD23 is incapable of translationally upregulating *Aurora-A* mRNA 5′-UTR-linked reporter gene (Supplementary Figure S6D). The possible mechanism leads to decreased activity in *exon2*-containing 5′-UTRs may result from the steric inhibitory effect of the secondary structure of *Aurora-A* mRNA 5′-UTR (Supplementary Figure S4B) and this is needed to be further investigated.

The effect of hnRNP Q1-dependent regulation of Aurora-A in promoting tumor-cell proliferation was demonstrated by cell-proliferation assay and colony-formation assay using hnRNP Q1/ΔRBD23 stable cells with or without exogeneous expressed Flag-Aurora-A. The results showed hnRNP Q1/ΔRBD23 stable cells have a more depressed proliferation ability than hnRNP Q1 stable cells; and when transfected with Flag-Aurora-A, the depressed proliferation ability was restored (Supplementary Figure S7A). The same result was obtained in colony-formation assay (Supplementary Figure S7B). These results support the description that hnRNP Q1-dependent regulation of Aurora-A may be involved in tumor growth or proliferation.

**Figure 4 fig4:**
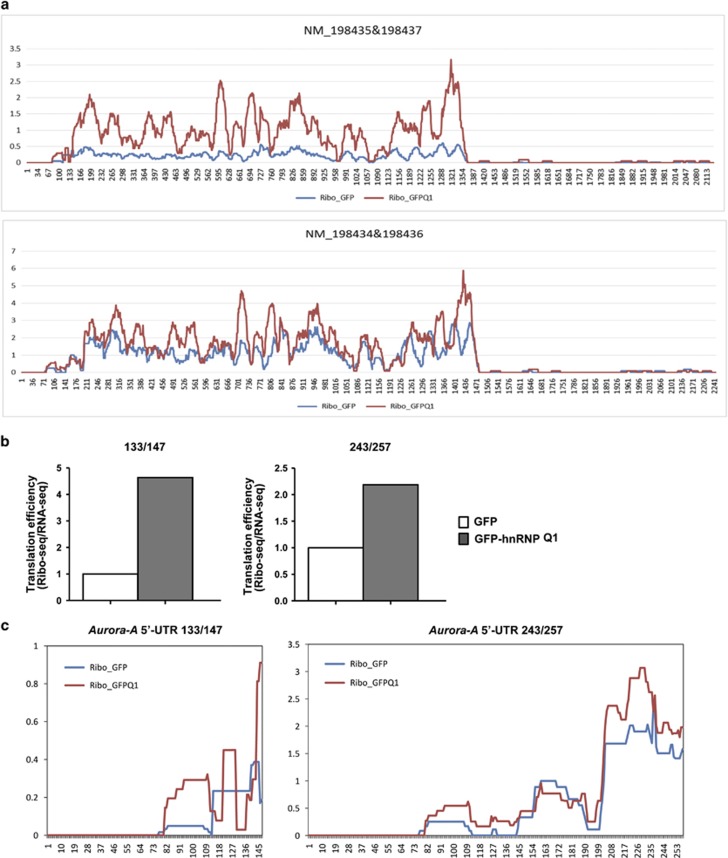
GFP-hnRNP Q1 enhances the translational efficiency of *Aurora-A* mRNA. (**a**) Ribosomal profiling of *Aurora-A* mRNA in GFP (blue) or GFP-hnRNP Q1 (red)-expressing cells. Total RNA was extracted from GFP or GFP-hnRNP Q1-expressing SW480 cells to collect the ribosome protection fragments for high-throughput sequencing. The *Aurora-A* mRNA short 5′-UTR isoforms (133/147, upper) and the long 5′-UTR isoforms (243/257, lower) are shown. The position of nucleotide is marked below. (**b**) Quantitative results showed the translational efficiency of *Aurora-A* mRNA isoforms from **a**[Fig fig4]. The reads from Ribo-seq were normalized by RNA-seq, and in which GFP-hnRNP Q1 was normalized by GFP vector control. (**c**) The enlarged picture of *Aurora-A* mRNA 5′-UTRs of **a**

The translational upregulation of *Aurora-A* mRNA by hnRNP Q1 was further demonstrated by S6-IP assay. The results showed the association between ribosome and *Aurora-A* mRNA is increased in GFP-hnRNP Q1-expressing cells and decreased in *hnRNP Q* siRNA-transfected cells (Figures 3d and e). Furthermore, the association between *Aurora-A* mRNA and eIF-4E, which is required for cap-dependent translation initiation, is increased in hnRNP Q1-expressing cells (Supplementary Figure S6E). To determine the translation regulation of *Aurora-A* mRNA by hnRNP Q1 comprehensively, the ribosomal profiling combined with whole-transcript high-throughput sequencing in hnRNP Q1-overexpressing cells was carried out. The translatome data suggested that ribosomes that scatter throughout the whole *Aurora-A* mRNA was increased in GFP-hnRNP Q1-overexpressing cells (Figure 4a). The translational efficiency of *Aurora-A* mRNA was enhanced when hnRNP Q1 was overexpressed; and the shorter *Aurora-A* 5′-UTR isoforms have higher efficiency than the longer isoforms (Figure 4b). In addition, ribosomes stalled on some specific regions of *Aurora-A* 5′-UTRs in hnRNP Q1-overexpressing cells, implying that those regions contain potential *cis*-regulatory element or structure; hnRNP Q1 enhances the recruitment of ribosomes to those regions of *Aurora-A* 5′-UTRs (Figure 4c). These results suggest hnRNP Q1 can upregulate the translation of *Aurora-A* mRNA, whereas GFP-hnRNP Q1/ΔRBD23 mutant loses the translational regulatory ability.

### HnRNP Q1 regulates *Aurora-A* mRNA translation in a cell cycle-dependent manner

**Figure 5:**
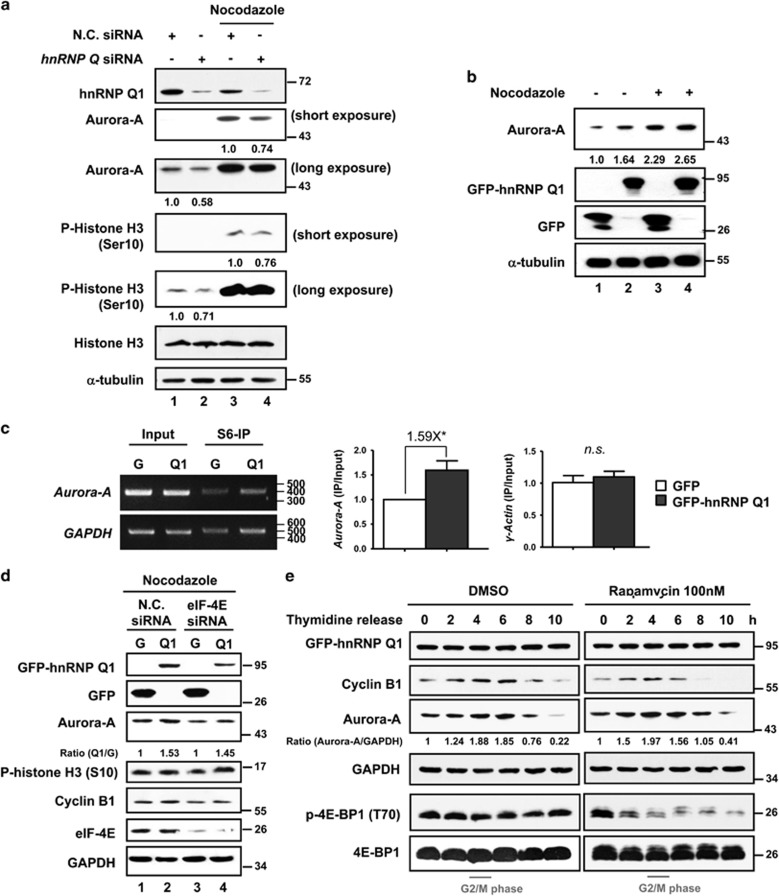
HnRNP Q1-enhanced Aurora-A protein expression is cell cycle dependent and occurs through cap-independent translational regulation. (**a**) SW480 cells transfected with control (NC siRNA) or *hnRNP Q* siRNA were treated with or without nocodazole. The whole-cell lysates were collected for western blot analysis using antibodies as indicated. The levels of Aurora-A (normalized by *α*-tubulin) and phospho-histone H3 (normalized by histone H3) were quantified as shown below. (**b**) Cells with GFP or GFP-hnRNP Q1 expression were treated with (+) or without (−) nocodazole to enrich the cell cycle mitotic stage. Total cell lysates were collected to perform western blot analysis using antibodies as indicated. (**c**) GFP (G) or GFP-hnRNP Q1 (Q1) expressing SW480 cells were synchronized by thymidine blocking and then released for 4 h to allow the cell cycle to enter into G2/M phase. The translation efficiency of *Aurora-A* mRNA was determined by S6-IP and the levels were evaluated by quantitative PCR as shown in right. *γ-Actin* was used as negative control. *P<0.05; n.s., no significance. (**d**) GFP (**G**) or GFP-hnRNP Q1 (Q1) stable cell line transfected with control (NC) or *eIF-4E* siRNA was treated with nocodazole and then collected for western blot analysis. The expression level of Aurora-A is displayed as ratio. (**e**) GFP-hnRNP Q1 stably expressing SW480 cells were synchronized by double thymidine treatment and then released for various time points in the presence of rapamycin or DMSO. Total cell lysates collected from different time points were collected for western blot using antibodies as indicated. Phospho-4E-BP1 (Thr70) serves as an indicator of the inhibitory effect of rapamycin for cap-dependent translation. Relative expression level of Aurora-A is displayed as ratio

Next, the regulatory behavior of hnRNP Q1 in *Aurora-A* mRNA translation during cell cycle progression was investigated. As shown in Figure 5a, the expression level of Aurora-A is increased in nocodazole-treated cells (compare lanes 1 and 3), whereas *hnRNP Q* siRNA treatment decreases the nocodazole-enhanced Aurora-A expression (compare lanes 3 and 4). This effect is observed in nocodazole-treated GFP-hnRNP Q1-overexpressing cells. Overexpressed GFP-hnRNP Q1 increases the level of Aurora-A at G2/M phase (Figure 5b, compare lanes 3 and 4). S6-IP assay further confirmed that GFP-hnRNP Q1 increases the translation efficiency of *Aurora-A* mRNA during mitotic stage (Figure 5c). These results support the idea that hnRNP Q1 can increase the translation of *Aurora-A* mRNA during cell cycle progression.

Because the cap-dependent translation is suppressed during mitosis,^21^ we further investigated whether the cell cycle-dependent translational regulation of *Aurora-A* mRNA by hnRNP Q1 is cap-dependent or not. To address it, eIF-4E was knocked down in GFP-hnRNP Q1 stable cells and then treated with nocodazole to determine the expression status of Aurora-A. The result indicated that hnRNP Q1-increased Aurora-A protein expression is not affected by the altered expression level of eIF-4E in nocodazole-treated cells (Figure 5d, compare the fold change of Aurora-A between lanes 1–2 and 3–4). A similar result was obtained in rapamycin-treated cells, in which the cap-dependent translation was inhibited^22^ (Figure 5e). In addition, the expression of GFP-hnRNP Q1 or endogenous hnRNP Q1 remains at a constant level throughout cell cycle progression, indicating the enhanced Aurora-A expression results from a translational upregulation by the constant expression level of hnRNP Q1 (Supplementary Figure S8A). The expression level of phosphorylated histone H3, which is an indicator of mitotic phase, is increased in GFP-hnRNP Q1 stably cells, whereas a GFP-hnRNP Q1/ΔRBD23 stable cell line did not have a similar effect (Supplementary Figure S8B).

### HnRNP Q1 regulates the IRES-dependent translation of *Aurora-A* mRNA in G2/M phase

**Figure 6:**
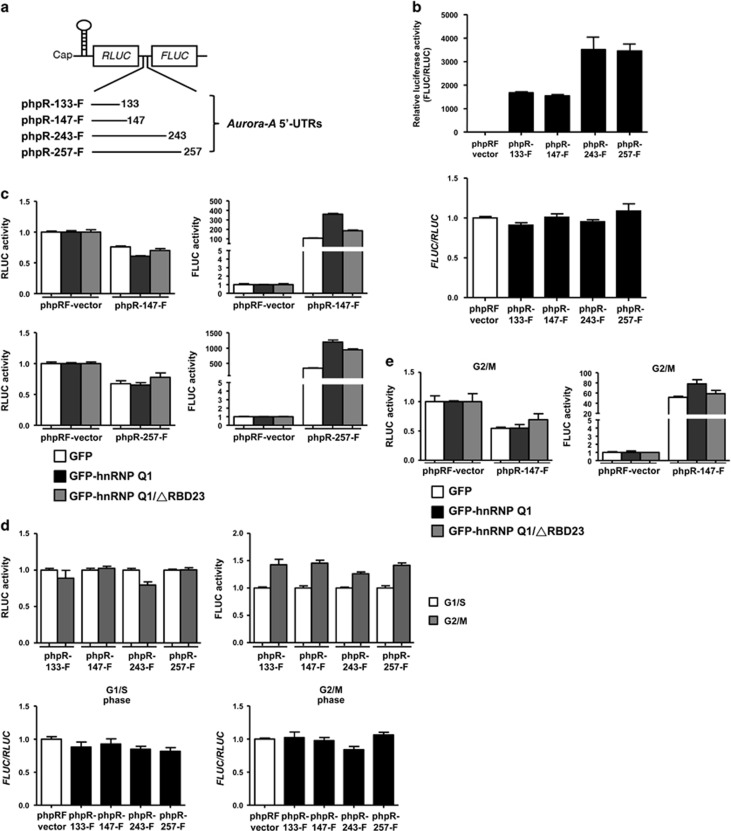
The IRES activity of *Aurora-A* 5′-UTRs is regulated by hnRNP Q1 and cell cycle-dependent. (**a**) Schematic illustration of the bicistronic reporter system (phpRF vector) of the four *Aurora-A* 5′-UTR isoforms. *RLUC*, *renilla luciferase*; *FLUC*, firefly *luciferase*. (**b**) HCT116 cells were transfected with different phpR-*Aurora-A* 5′-UTRs-F reporter plasmids, and then their IRES activity was determined by *in vivo* translation assay and shown as ratio of FLUC to RLUC (upper). The expression level of *renilla luciferase* mRNA and firefly *luciferase* mRNA are shown as ratio below. (**c**) The IRES activity of *Aurora-A* 5′-UTR 147 nt or 257 nt isoforms in GFP, GFP-hnRNP Q1 or GFP-hnRNP Q1/ΔRBD23-expressing HCT116 cells was determined by measuring the RLUC and FLUC activities. phpRF vector was used as a negative control of hnRNP Q1. (**d**) HCT116 cells transfected with different phpR-*Aurora-A* 5′-UTRs-F reporter plasmids were synchronized at G1/S phase or G2/M phase by 2 mM thymidine or 50 ng/ml nocodazole, and then their IRES activity was determined as described in **c**. The expression levels of *renilla luciferase* mRNA and firefly *luciferase* mRNA in G1/S and G2/M phase were evaluated by RT-qPCR and shown as ratio in **b** (lower). (**e**) HCT116 cells co-transfected with GFP-hnRNP Q1 (wt or ΔRBD23) and phpR-147- F were synchronized at G2/M phase by nocodazole, and then the IRES activity was determined as described above in **c**

Dobson *et al.*^23^ have demonstrated that the *Aurora-A* 5′-UTR contains IRES activity, which may be responsible for *Aurora-A* mRNA translation during cell division. However, which *Aurora-A* mRNA 5′-UTR variants respond to IRES-dependent translation and which *trans*-acting factors (ITAFs) regulate *Aurora-A* mRNA 5′-UTR IRES activity remain unclear. To address these questions, the IRES activity of the four *Aurora-A* mRNA 5′-UTR isoforms was checked by bicistronic reporter assay, and a hairpin structure was inserted ahead of the *Aurora-A* mRNA 5′-UTRs to block the cap-dependent translation (Figure 6a and Supplementary Figure S9A). The results showed that all of the four *Aurora-A* mRNA 5′-UTR variants exhibited IRES activity, and the *exon2*-containing *Aurora-A* mRNA 5′-UTR variants had stronger IRES activities than the *exon1*-only variants (Figure 6b and Supplementary Figure S10A). The cryptic promoter or splicing of the four phpR-*Aurora-A* 5′-UTR-F plasmids was excluded (Supplementary Figures S9B–D). Moreover, the *Aurora-A* 5′-UTR IRES activity was enhanced by GFP-hnRNP Q1 but only moderately increased by GFP-hnRNP Q1/ΔRBD23 (Figure 6c and Supplementary Figure S10B).

The IRES activities of all four *Aurora-A* mRNA 5′-UTR variants were elevated in G2/M phase compared with G1/S phase (Figure 6d and Supplementary Figure S10C). GFP-hnRNP Q1 increased the *Aurora-A* 5′-UTR IRES activity in G2/M phase, but GFP-hnRNP Q1/ΔRBD23 only exhibited a partial effect (Figure 6e and Supplementary Figure S10D). Neither GFP-hnRNP Q1 nor GFP-hnRNP Q1/ΔRBD23 alters the basal reporter activity of phpRF vector (Supplementary Figure S10E). These results suggest that hnRNP Q1 is a potential ITAF that targets the *Aurora-A* 5′-UTR to regulate its IRES-mediated translation during mitosis.

### HnRNP Q1 promotes tumorigenicity

**Figure 7:**
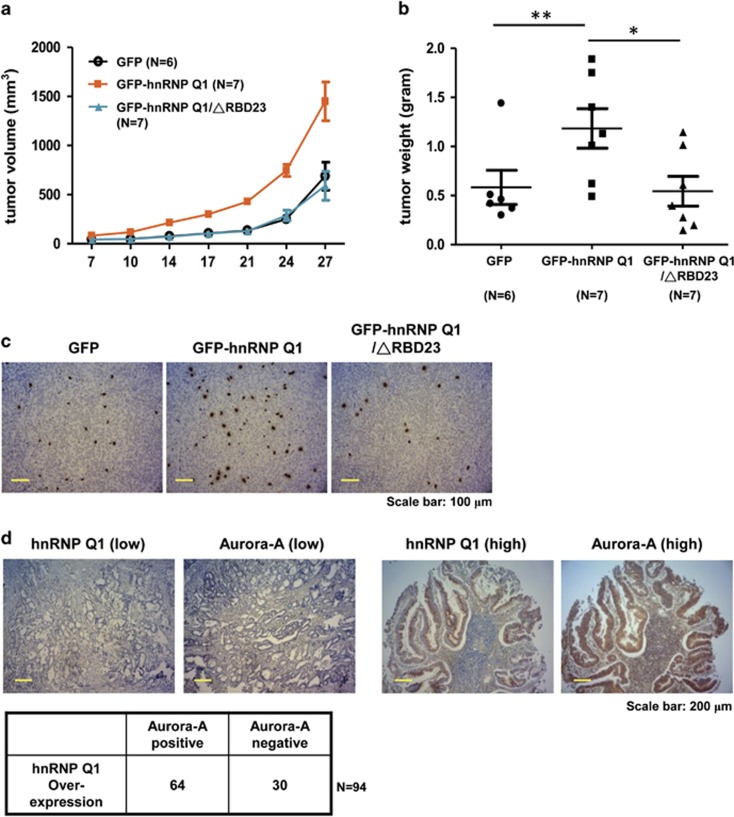
Translational upregulation of *Aurora-A* by hnRNP Q1 contributes to tumor growth, and the expression of Aurora-A is positively correlated with hnRNP Q1 in colorectal cancer tissues. (**a** and **b**) SW480 cells stably expressed GFP, GFP-hnRNP Q1 or GFP-hnRNP Q1/ΔRBD23 were subcutaneously injected into NOD-SCID mice. The tumor volume (**a**) and tumor weight (**b**) are shown; *P<0.05 and **P<0.01. (**c**) The representative images show the IHC result of phospho-histone H3/serine10 in the GFP, GFP-hnRNP Q1 or GFP-hnRNP Q1/ΔRBD23-bearing tumors. (**d**) The representative image of IHC assay showed the positive correlation of Aurora-A and hnRNP Q1 in human colorectal cancer tissues. A total of 94 colorectal cancer tissues were analyzed

For further evaluating the role of hnRNP Q1 in cancer, a xenograft animal model was used to address the tumorigenecity of hnRNP Q1. The results showed that GFP-hnRNP Q1 stable cells can promote greater tumor growth than GFP-expressing cells (Figures 7a, b, and Supplementary Figure S11A). Interestingly, cell with GFP-hnRNP Q1/ΔRBD23 expression, which loses the *Aurora-A* 5′-UTR binding ability, has the same effect as GFP-expressing cells (Figures 7a, b, and Supplementary Figure S11A). Expression level of Aurora-A in GFP, GFP-hnRNP Q1 and GFP-hnRNP Q1/ΔRBD23-expressing tumors was checked by western blot analysis (Supplementary Figure S11B). Immunohistochemistry (IHC) showed that expression of phospho-histone H3/serine10 is much higher in GFP-hnRNP Q1-bearing tumors than GFP or GFP-hnRNP Q1/ΔRBD23-expressing tumors (Figure 7c). In addition, the expression pattern of Aurora-A and hnRNP Q1 is positively correlated in human colorectal cancer (CRC) tissues (Figure 7d).

## Discussion

HnRNP Q1 has been shown to bind to the AU-rich region,^3, 16^ which is widespread in *Aurora-A* 5′-UTR *exon1*. In the present study, biotin pull-down assay revealed the *Aurora-A* mRNA 5′-UTR *exon1* or *exon1* variant presented a higher binding ability of hnRNP Q1 than the *exon1* plus *exon2* variants (Figures 2a and b), and the *cis*-interacting element for hnRNP Q1 targeting site is mapped in the fore and middle regions of *Aurora-A* mRNA 5′-UTR 147 nt (Supplementary Figure S4C). Meanwhile, some ribosomes stalled on the nucleotides between 81 and 111 of all *Aurora-A* 5′-UTRs, which is the middle region of *Aurora-A* mRNA 5′-UTR 147 nt; and ribosomes have an increased occupancy in that region when hnRNP Q1 was overexpressed (Figure 4c, Supplementary Figure S4). By structure prediction, we found this region can form a stable hairpin loop independently (Supplementary Figure S4B). The association between hnRNP Q1 and this region was further confirmed by *in vitro* biotin pull-down assay (147-B, Supplementary Figures S4C). Even hnRNP Q1 can associate with the 5′-end of 5′-UTR (147- A) *in vitro* (Supplementary Figure S4C), however, this region cannot form an independent hairpin structure in full-length 5′-UTR (Supplementary Figure S4B). Therefore, we supposed that the targeting site of hnRNP Q1 may limit in the middle region of *Aurora-A* 5′-UTR. Whether the AU-rich sequence and the RNA secondary structure of this region both contribute to the hnRNP Q1 recognition needs to be further verified.^24^ In addition, our result showed that the existence of RBD1 reduces the RNA-binding ability of RBD2 and RBD3 (Figure 2e). This effect may result from the steric effect of RBD1, which prohibits RBD2/3 to target *Aurora-A* mRNA 5′-UTR; alternatively, hnRNP Q1 targeting *Aurora-A* 5′-UTRs may be controlled by posttranslational modification that causes its protein structure to change, exposing the central region (RBD2 and RBD3) to increase the RNA-binding ability.^25, 26^

Although the RBD1 and RGG box of hnRNP Q1 cannot bind to *Aurora-A* 5′-UTRs (Figure 2e), the possibility that these two RNA-binding domains might recognize other consensus sequences or associate with other hnRNPs to target *Aurora-A* mRNAs still cannot be excluded. The composition of multiple RNA-binding domains and auxiliary domains, such as the RGG box, is a common characteristic of the hnRNP family. The various binding affinities of each domain might contribute to the selection of the specific target mRNAs under different conditions.^1^

Our results indicated that hnRNP Q1 interacts with *Aurora-A* mRNA 5′-UTR through the RBD23 domains of hnRNP Q1 (Figures 2e–g). Interestingly, overexpressed GFP-hnRNP Q1/ΔRBD23 changes the cytosolic distribution as GFP-hnRNP Q1 full-length protein does, and instead of nuclear localization where it is far from the to-be regulated mRNA (Supplementary Figure S3), for example the *Aurora-A* mRNA. Therefore, the changed subcellular localization of GFP-hnRNP Q1/ΔRBD23 may result in the impaired translational upregulation of *Aurora-A* mRNA.

In the bicistronic reporter system, the IRES activities are higher in the longer *Aurora-A* 5′-UTR variants than the shorter variants (Figure 6b and Supplementary Figure S10A). In contrast to the shorter *Aurora-A* 5′-UTRs showing a higher translational activity to hnRNP Q1 in the monocistronic reporter system (Supplementary Figure S6B); the *exon2* in the longer *Aurora-A* 5′-UTR variants may offer additional *cis*-elements or contribute to form a stable secondary structure which benefit IRES-mediated translation. In addition, hnRNP Q1 has been demonstrated to associate with multiple ribosomal subunits,^8^ which might explain why we found hnRNP Q1 can also enhance housekeeping gene *GAPDH* translation (Figure 3d and Supplementary Figure S6E). We suggest that hnRNP Q1 could be a widespread translational regulator.

Furthermore, we found that both mRNA and protein levels of hnRNP Q1 are elevated in the tumor part of CRC compared with adjacent normal tissues (Figure 7d and data not shown). A previous report indicated that the protein stability of hnRNP Q protein is maintained by Galectin-3 in colon cancer;^18^ however, the increased mRNA level in tumors is still unclear. In colorectal cancer, the region of chromosome 6q14, where *HNRNPQ* (symbol: *SYNCRIP*) is located, has no alternation or deletion, indicating that the increased hnRNP Q may not result from gene amplification.^27, 28^ It is possible that the increased *hnRNP Q* mRNA in tumors might be due to mechanisms involving transcriptional upregulation or post-transcriptional regulation, such as miRNA.^29^ The content of cancer cells might contribute additional signals in the posttranslational modification of hnRNP Q1 for its retention in the cytoplasm and its function in the translational regulation of mRNAs.^26^

Our previous work demonstrated that the MEK/ERK and Akt/mTOR pathways participate in EGF-induced *Aurora-A* translational upregulation in a cap-dependent manner.^19^ The current study showed that, during mitosis, the translational regulation of *Aurora-A* mRNA by hnRNP Q1 is not affected by eIF-4E siRNA or rapamycin treatment (Figures 5d and e), supporting the IRES-mediated translational upregulation and implying the involvement of another pathway. Identifying the upstream signaling pathway involved in hnRNP Q1-regulated *Aurora-A* mRNA IRES-mediated translation is an important issue for finding potential targets of cancer therapy.

## Materials and Methods

### Patient specimens

Studies of clinical specimens have been conducted according to the Helsinki Declaration and the laboratory protocol has been approved by the institutional review board of National Cheng Kung University Hospital (A-ER-100-403).

### Next-generation high-throughput sequencing (RNA-seq) and analysis

All the procedures were performed according to Illumina's protocol. Library constructions were estabilished by TruSeq RNA Sample Prep Kits v2 on Solexa platform (Illumina, San Diego, CA, USA). The RNA sequences were determined by using sequencing-by-synthesis technology via the TruSeq SBS Kit. For RNA-seq analysis, the sequences went through a filtering process to obtain qualified reads.^30^ The reads were analyzed by using TopHat/Cufflinks^31^ for gene expression estimation. The levels of genes were calculated as FPKM (Fragments Per Kilobase of transcript per Million mapped reads). The reference genome and gene annotations were obtained from Human_hg19 (GRCh37.69) of Ensembl database. The data collection and analysis were performed by Welgene Biotech Co., Ltd (Taipei, Taiwan). NGS results were submitted to GEO (Gene Expression Omnibus) with an accession number GSE76457.

### Biotin pull-down assay

The biotin-labeled RNA probe was synthesized by *in vitro* transcription system (Promega, Madison, WI, USA) according to the manufacturer's instructions. Briefly, *Aurora-A* 5′-UTR was transcribed by T7 RNA polymerase (Promega) and incorporated with biotin-14-CTP (Invitrogen, Carlsbad, CA, USA) at 37 °C for 1.5 h. After treatment with RQ1 DNase (Promega), the biotin-labeled RNA probe was purified using the illustra MicroSpin G-25 Columns (GE Healthcare, Chicago, IL, USA). Total cell lysates collected by RIPA buffer were pre-cleaned by streptavidin beads (Sigma, St. Louis, MO, USA) at 4 °C for 1 h. For pull-down assay, the total cell lysates were diluted with 5X EMSA buffer (50 mM Hepes [pH 8], 250 mM KCl, 10 mM MgCl_2_, 5 mM DTT and 25% glycerol) including 0.5 mg/ml tRNA, 0.7 mg/ml heparin, 100 unit/ml RNase inhibitor, protease inhibitors and biotin-labeled RNA probes. After incubating at 4 °C for 2 h, the mixtures were subjected to UV-crosslinking (120 mJ/cm^2^) for 5 min three times, and then streptavidin beads were added to rotate at 4 °C for 1 h. The beads were washed by the wash buffer (10 mM Hepes (pH 8), 40 mM KCl, 3 mM MgCl_2_, 2 mM DTT, 5% glycerol, 0.5% SDS and 2% NP-40) three times, followed by western blot analysis.

### Degradation assay

SW480 cells were transfected with GFP or GFP-hnRNP Q1 for 24 h and then treated with cycloheximide (200 *μ*g/ml) (Sigma) for different time periods. The cell lysates were collected by RIPA buffer and analyzed by western blot. The expression level of Aurora-A protein was normalized by *α*-tubulin and displayed the degradation curve at each time point.

### *In vivo* translation assay

The cells were co-transfected with *Aurora-A* 5′-UTR-pGL3 plasmids and pRL-TK vector for 12 h and then collected by 1 × passive lysis buffer (Promega). The activity of firefly and *renilla* luciferase was measured according to the protocol of the Dual-Luciferase Reporter Assay System (Promega) and detected by Minilumat LB 9506.

### IRES reporter assay

The cells were transfected with *Aurora-A* 5′-UTR-phpRF plasmids for 24 h and collected by 1 × passive lysis buffer (Promega). The activity of firefly and *renilla* luciferase activity was measured by Dual-Luciferase Reporter Assay System (Promega) as described above.

### Ribosomal protein S6 immunoprecipitation assay

The cell lysates for S6-IP were collected by RNA-IP buffer (10 mM Hepes (pH 8), 100 mM KCl, 5 mM MgCl_2_, 1% Triton X-100, 0.5% sodium deoxycholate, 100 unit/ml RNase inhibitor, 100 *μ*g/ml cycloheximide). The obtained cell lysates were pre-cleaned by protein A beads at 4 °C for 1 h, and then immunoprecipitated by anti-ribosomal protein S6 antibody (Santa Cruz) for 2 h, followed by incubating with protein A beads for a further 1 h at 4 °C. The S6-IP beads were washed with RNA-IP buffer three times, and RNAs were extracted by Trizol (Invitrogen). The S6-bounded mRNAs were analyzed by RT-PCR or RT-qPCR as indicated in Supplementary Materials and Methods.

### RNA-immunoprecipitation assay

The cells were collected and lysed by RNA-IP buffer (10 mM HEPES (pH 8), 40 mM KCl, 3 mM MgCl_2_, 2 mM DTT, 5% glycerol, 0.5% sodium deoxycholate, 100 unit/ml RNase inhibitor and protease inhibitors). The cell lysates were immunoprecipitated by anti-GFP antibody (JL-8, Clontech, Mountain View, CA, USA) or anti-eIF-4E antibody (P-2, Santa Cruz, Santa Cruz, CA, USA) for 2 h at room temperature and then incubated with 1 mg/ml tRNA pre-cleaned protein A/G beads for a further 2 h at room temperature. The bead-bound IP complex was washed with RNA-IP buffer three times and RNA was extracted by TRIsure (Bioline, Taunton, MA, USA) and analyzed by RT-PCR or real-time PCR.

### Recombinant proteins preparation and Biacore surface plasmon resonance analysis

The generation of GST-hnRNP Q1 fragments and the production of GST fusion proteins were conducted as described previously.^32^ Biotin-labeled RNA probe was synthesized using an *in vitro* transcription kit according to the manufacturer's instructions. (Promega). For surface plasmon resonance (SPR) analysis, the RNA probes were diluted in HBS-N buffer (final 10 mM HEPES (pH 7.4), 3 mM EDTA, 300 mM NaCl, 0.005% P20, in DEPC water) and injected into the sensor chip SA (GE Healthcare) at a rate of 10 *μ*l/min for 1 min to immobilize on the chip. After immobilization, GST fusion proteins were diluted in HBS-N buffer to different concentrations, injected into the chip in a rate of 10 *μ*l/min for 3 min, and then washed with HBS-N buffer for 7 min to detect the RNA–protein interaction on the chip by evaluating the response unit (RU). The chip was washed with regeneration buffer (3 M NaCl) to disrupt the RNA–protein interaction and rescue the probe-binding ability between each cycle of experiments.

### Statistical analysis

All the experiments were repeated at least three times, and the error bar was shown as mean±S.E.M. Student's *t*-test was used for analyzing the result of experiments in this paper. *P*-value <0.05 was regarded as significant.
