# Supplementary material for: Translational upregulation of Aurora-A by hnRNP Q1 contributes to cell proliferation and tumorigenesis in colorectal cancer
Source: Cell Death Dis. 2017 Jan 12;8(1):e2555–. doi: 10.1038/cddis.2016.479 (PMC5386382; doi:10.1038/cddis.2016.479)

Supplementary Figure S1

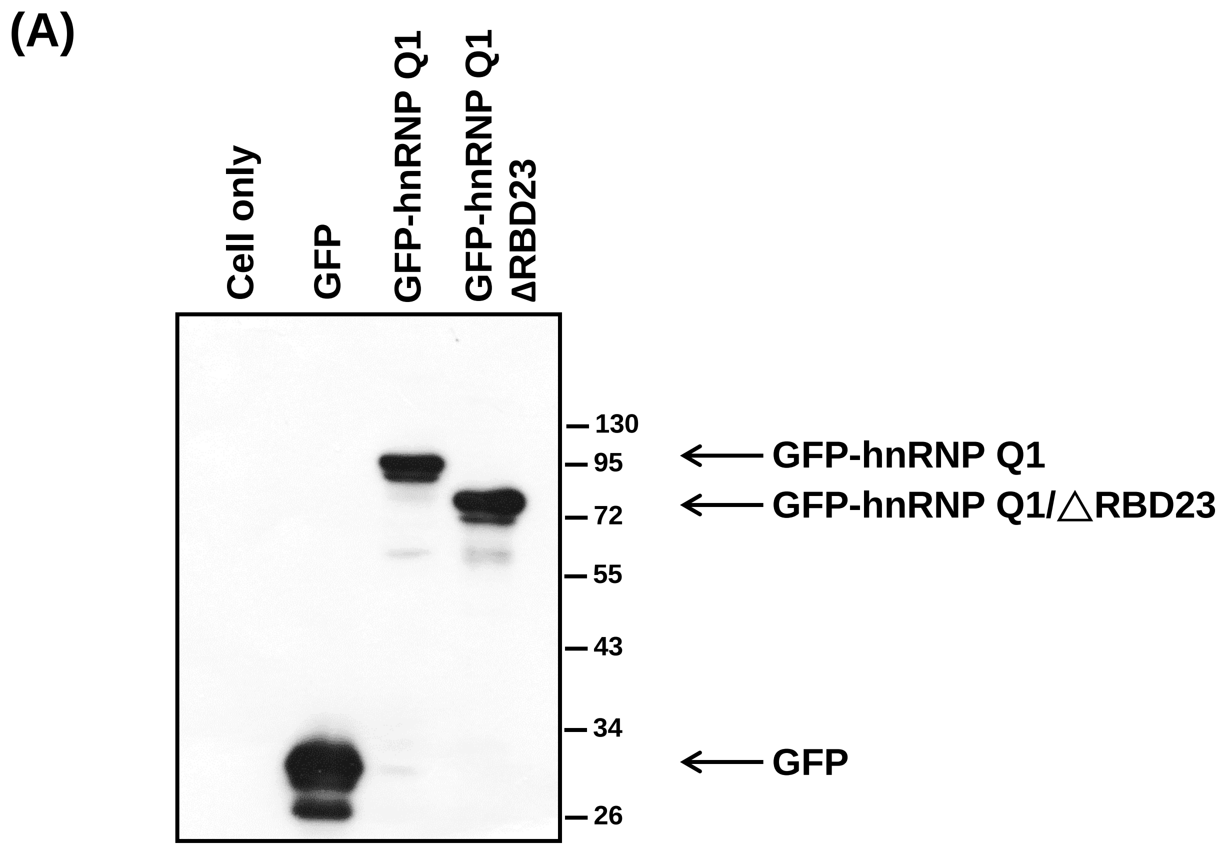

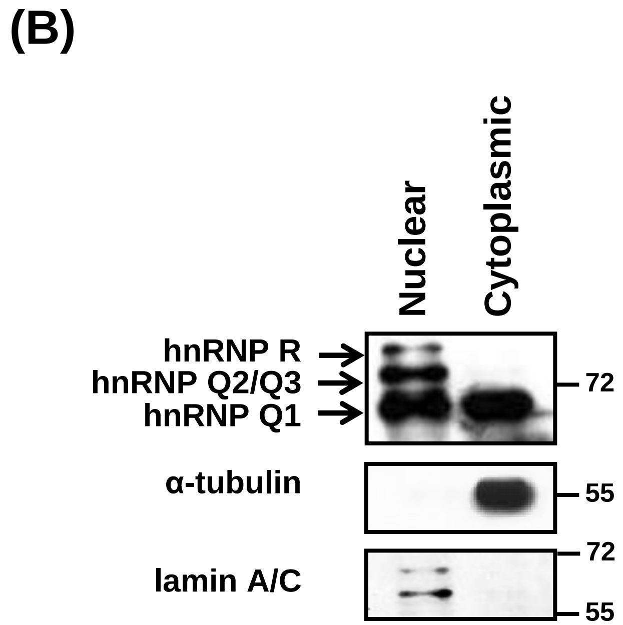

Supplementary Figure S2

(A)

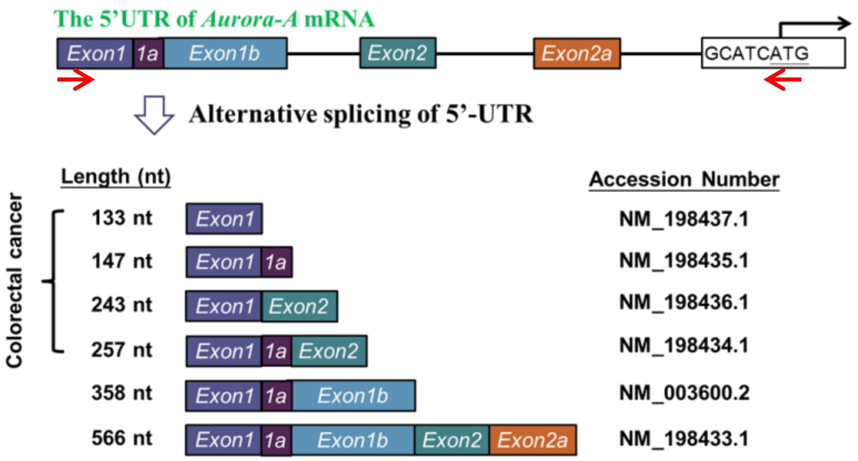

(B)

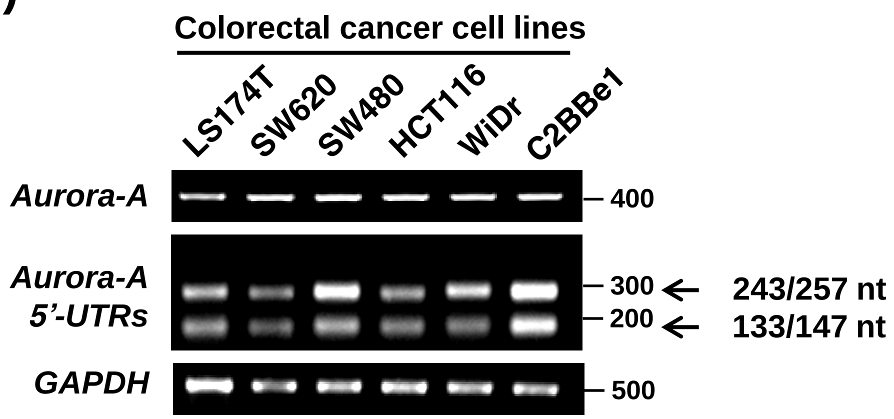

(C)

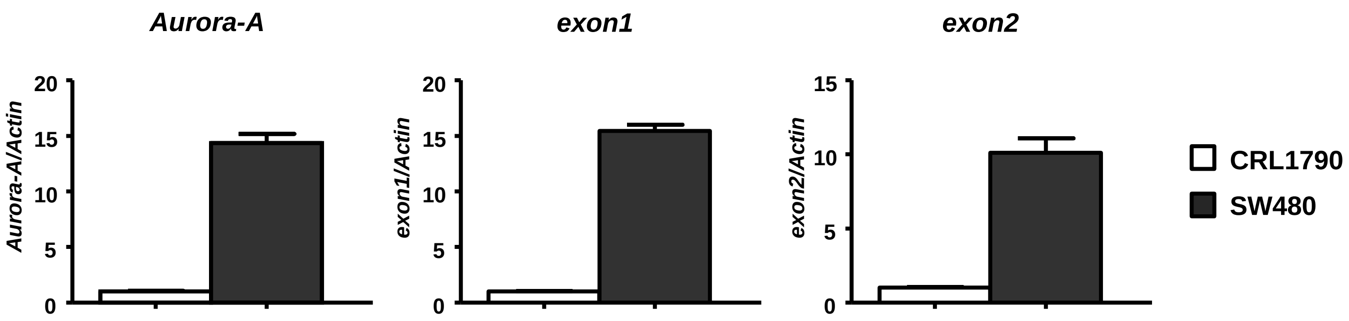

Supplementary Figure S2

(D)

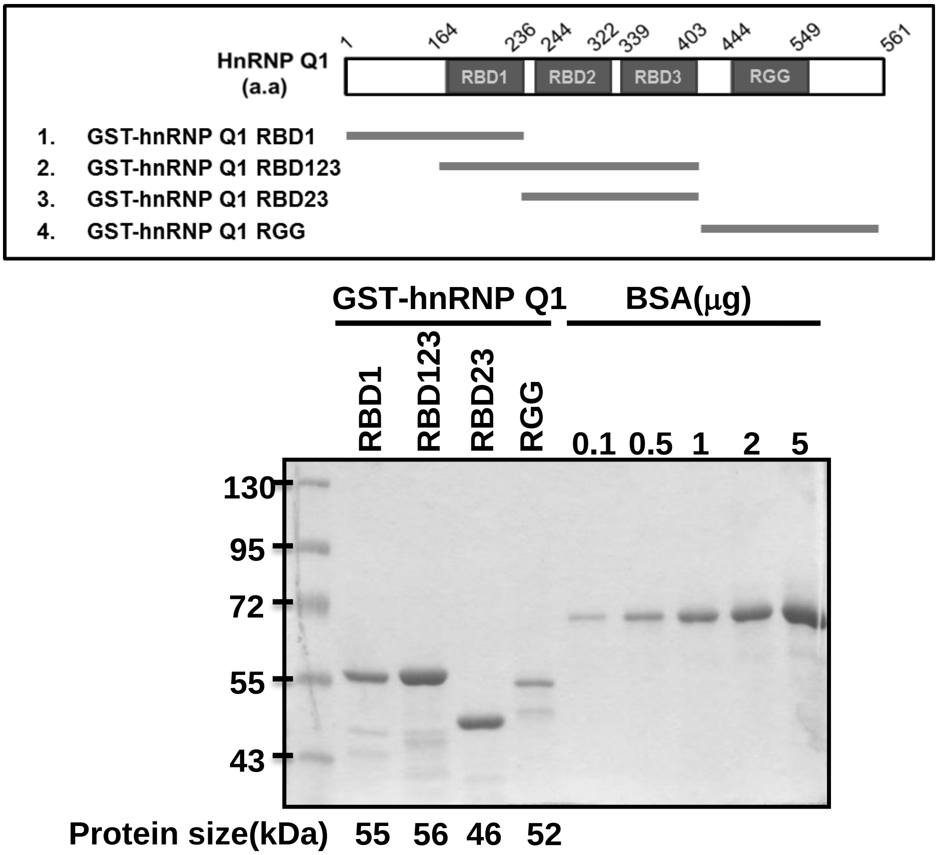

Supplementary Figure S3

GFP DAPI

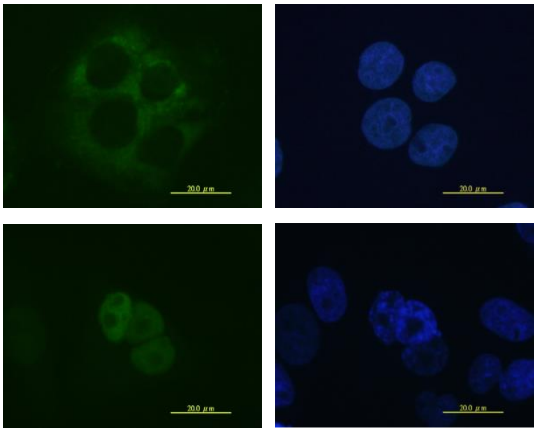

SW480
GFP-hnRNP Q1

SW480
GFP-hnRNP Q1/ΔRBD23

Scale bar: 20.0 μm

Supplementary Figure S4

(A)

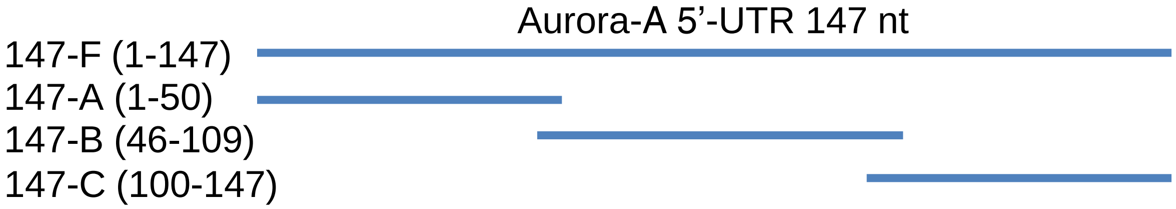

(B)

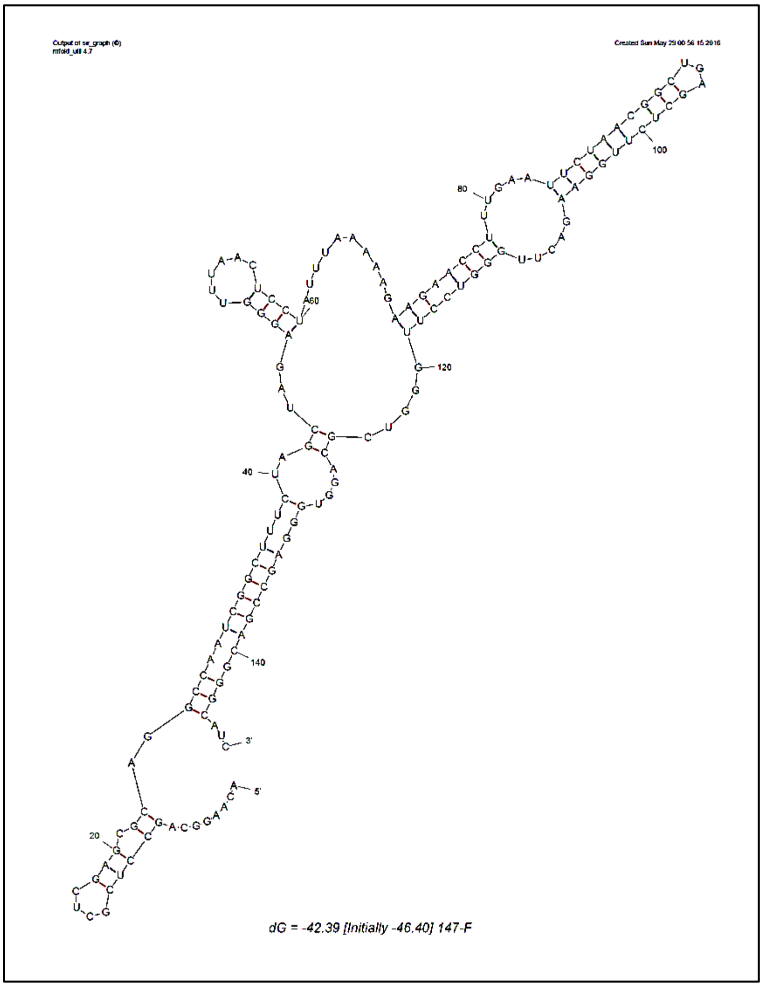

(C)

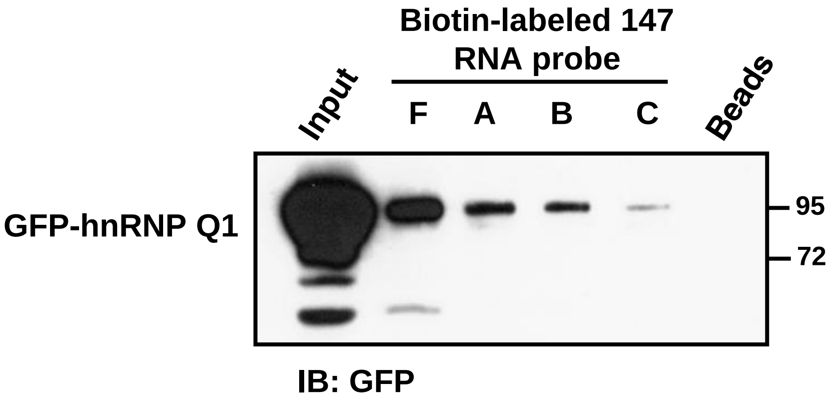

Supplementary Figure S5

(A)

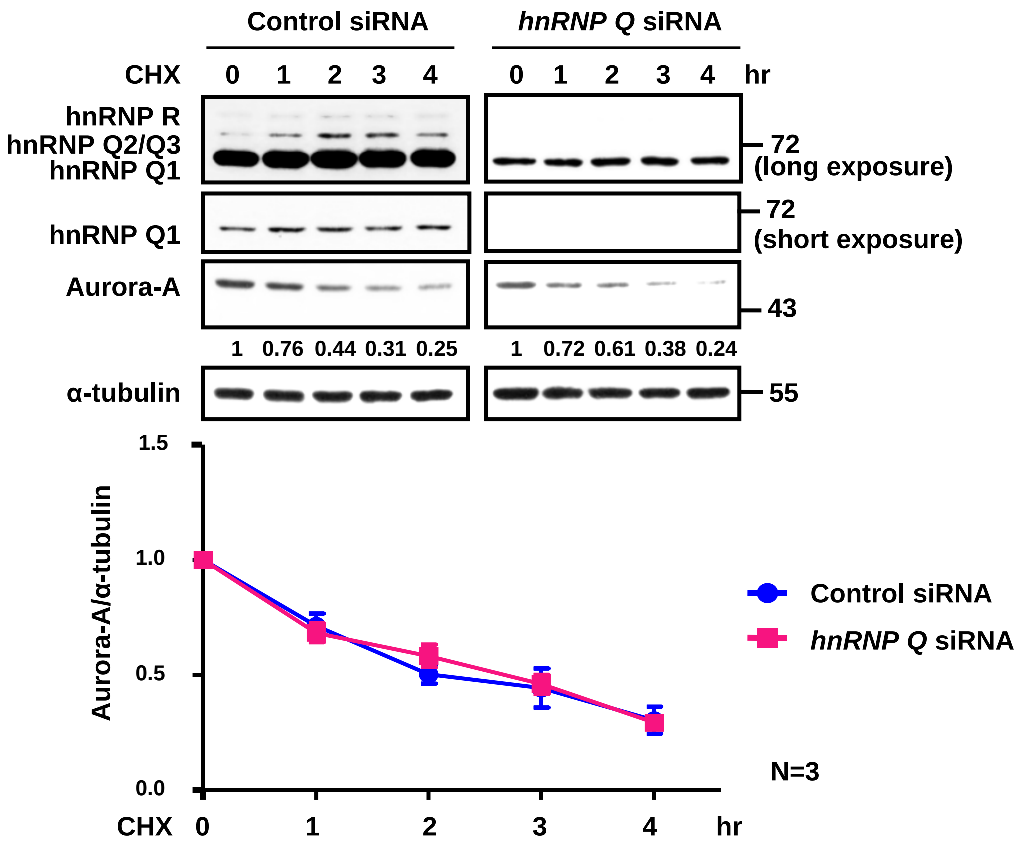

(B)

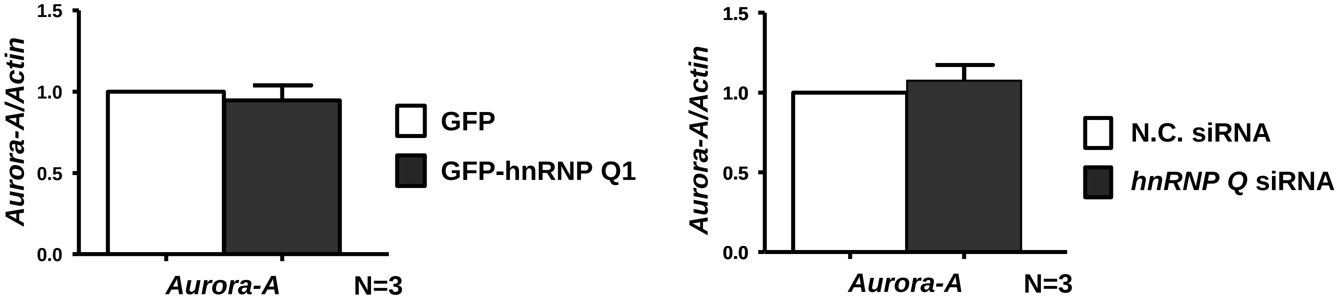

(C)

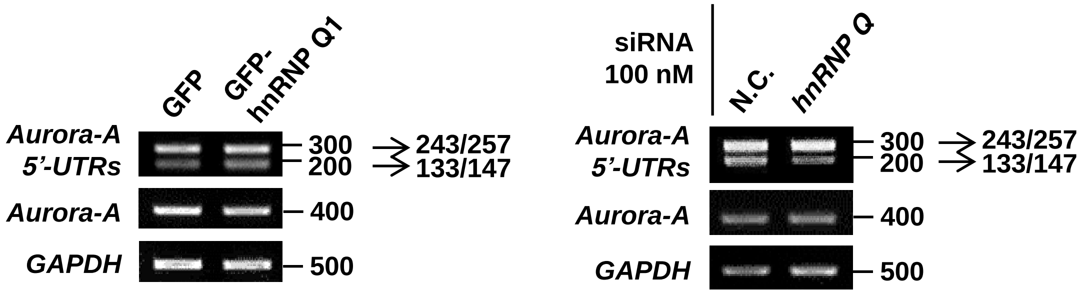

Supplementary Figure S6

(A)

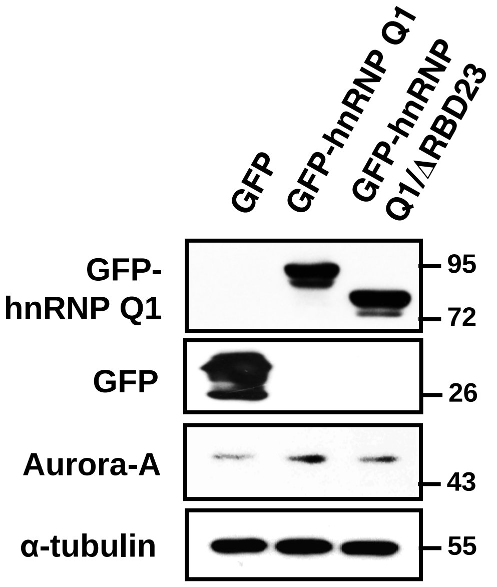

(B)

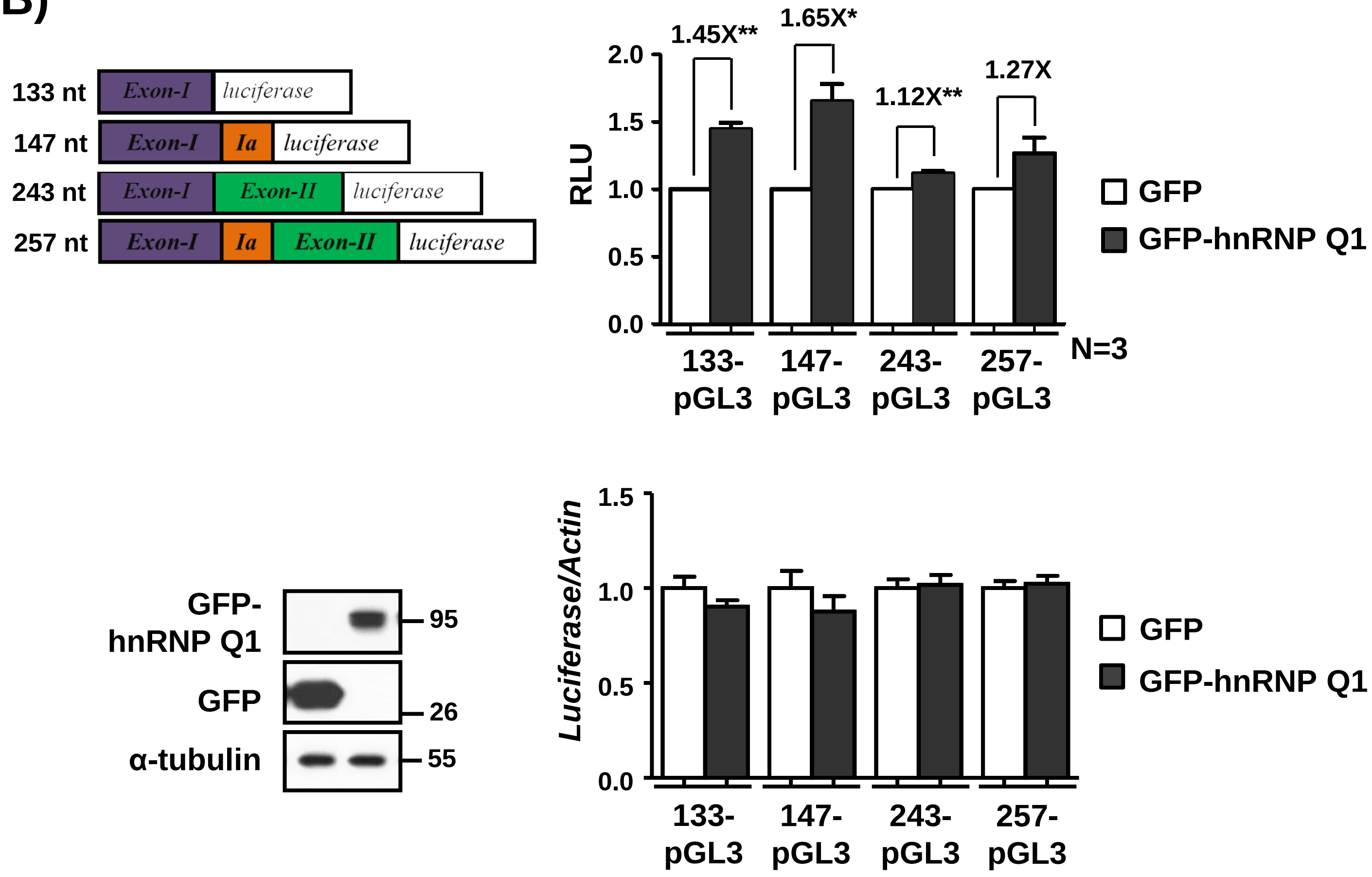

Supplementary Figure S6

(C)

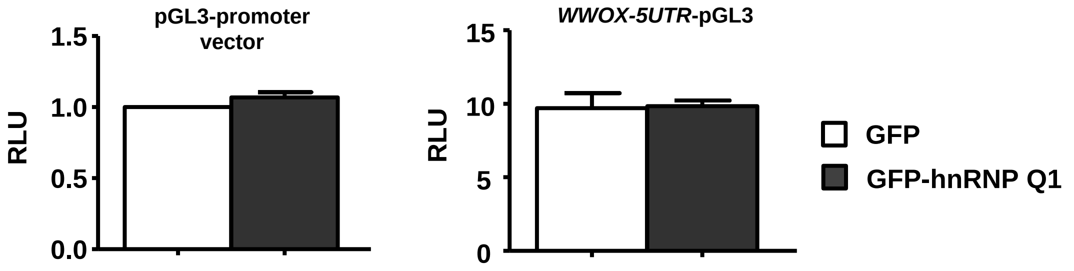

(D)

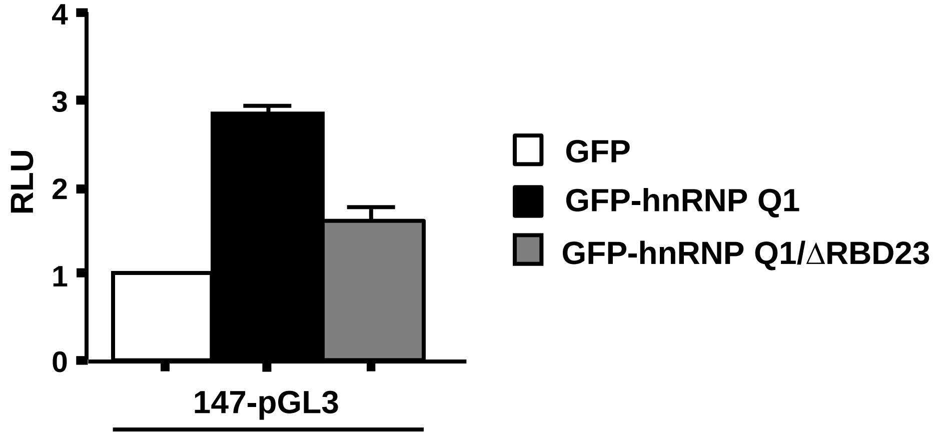

(E)

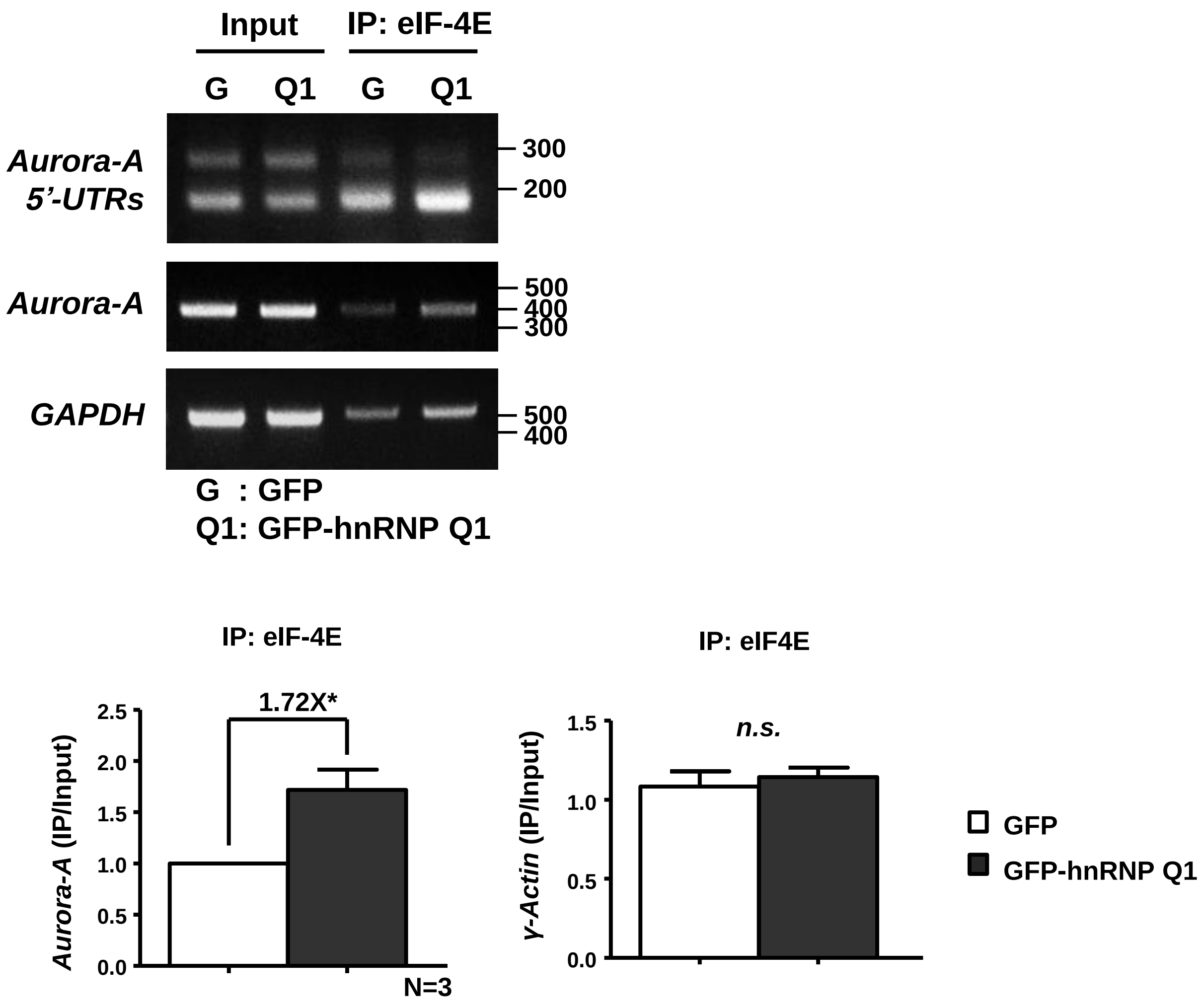

Supplementary Figure S7

(A)

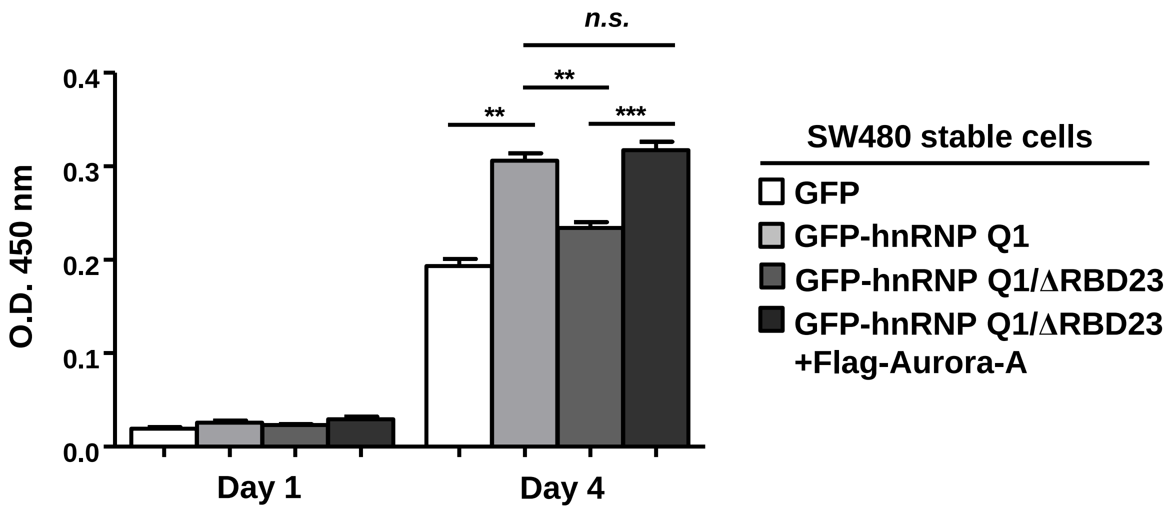

(B)

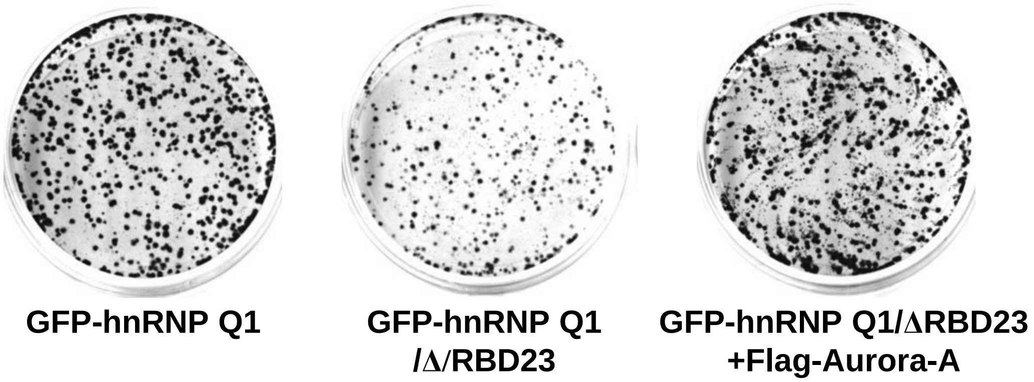

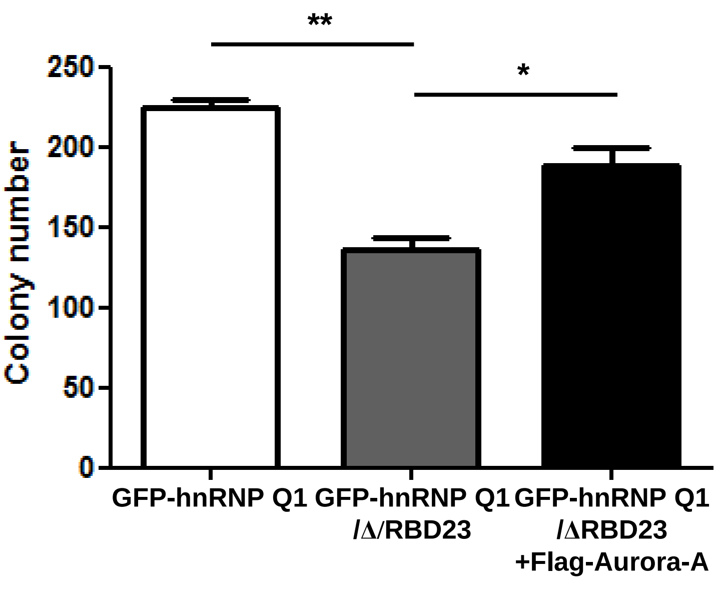

Supplementary Figure S8

(A)

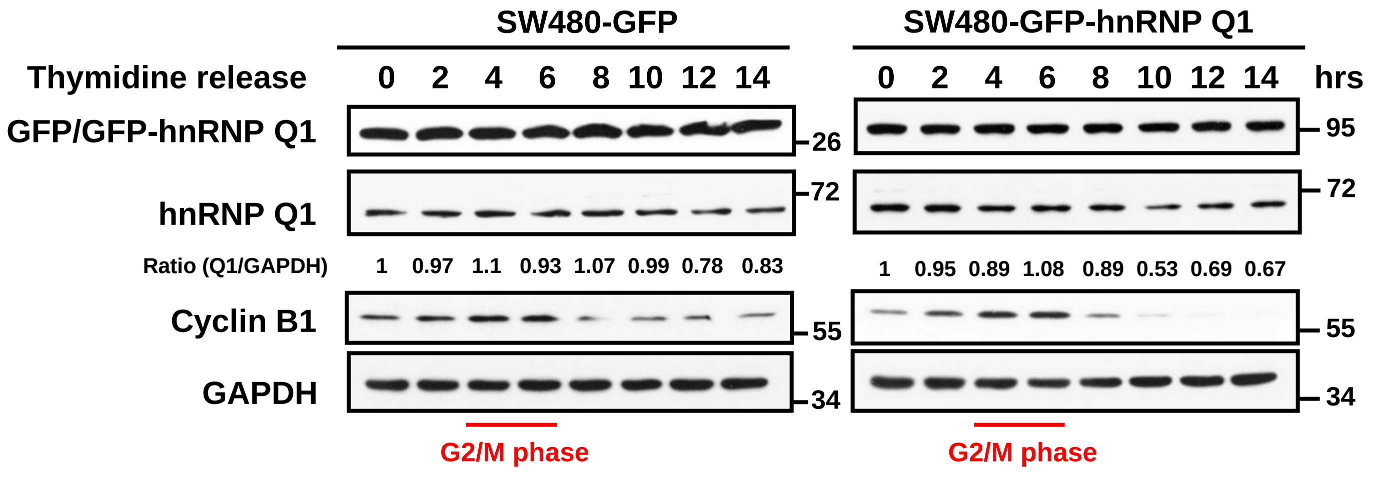

(B)

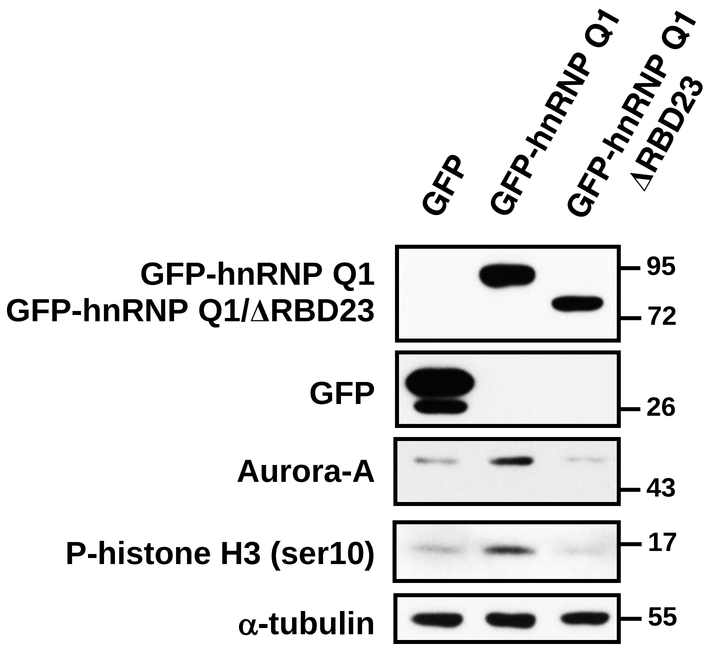

Supplementary Figure S9

(A)

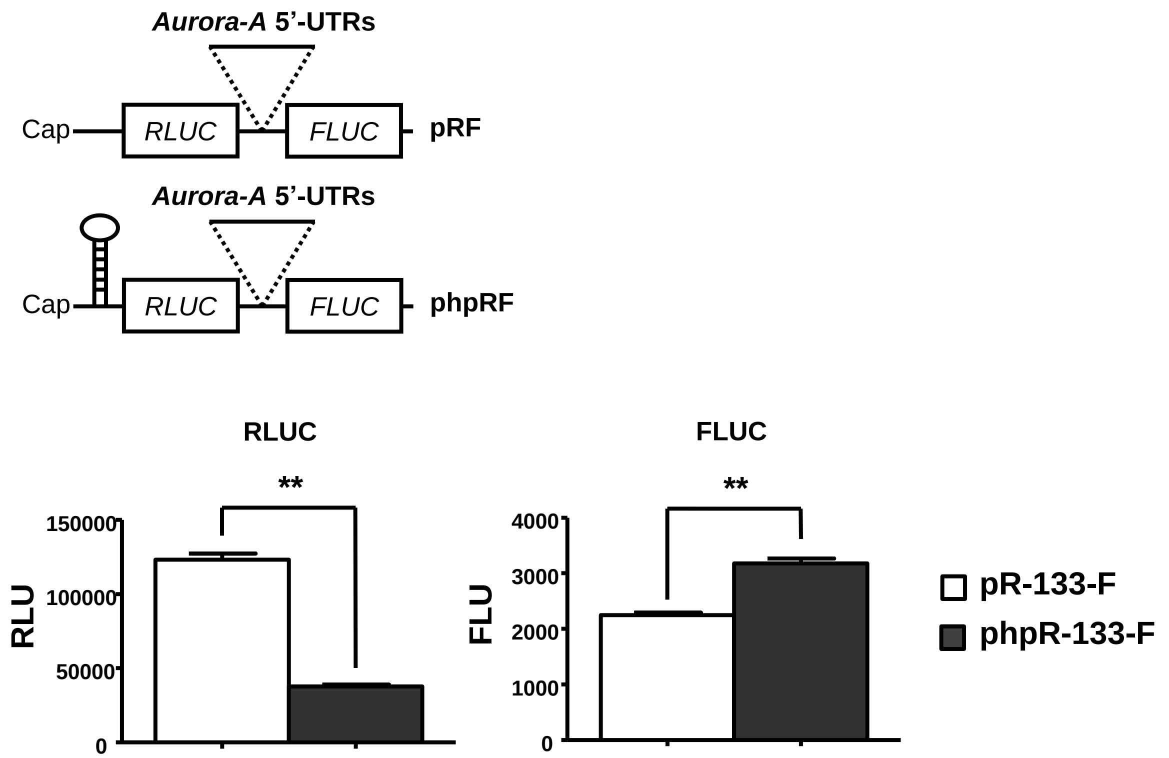

(B)

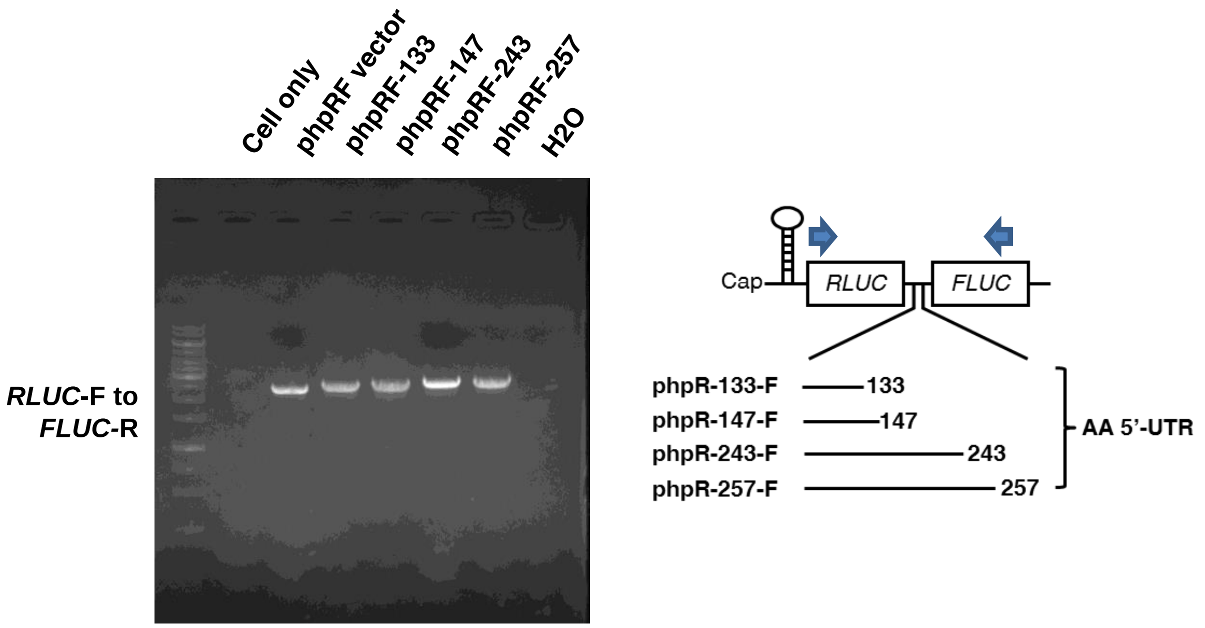

Supplementary Figure S9

(C)

phpR-133-F

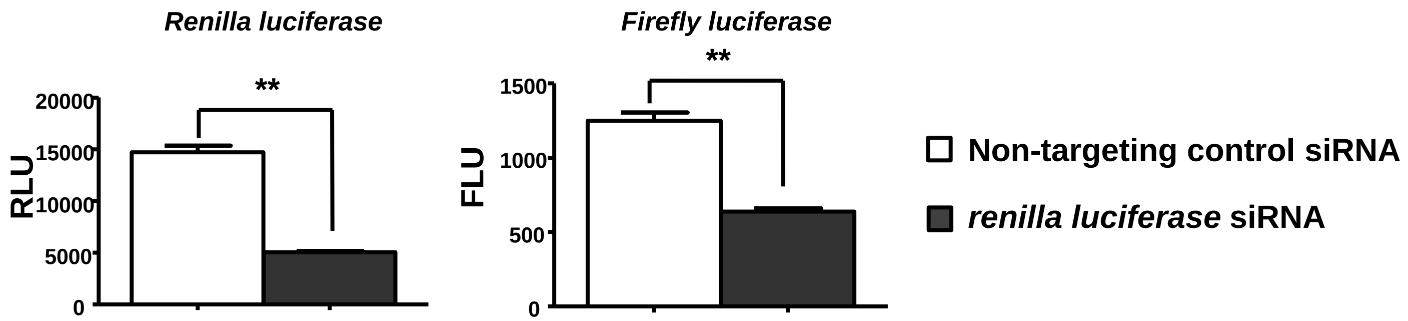

phpR-147-F

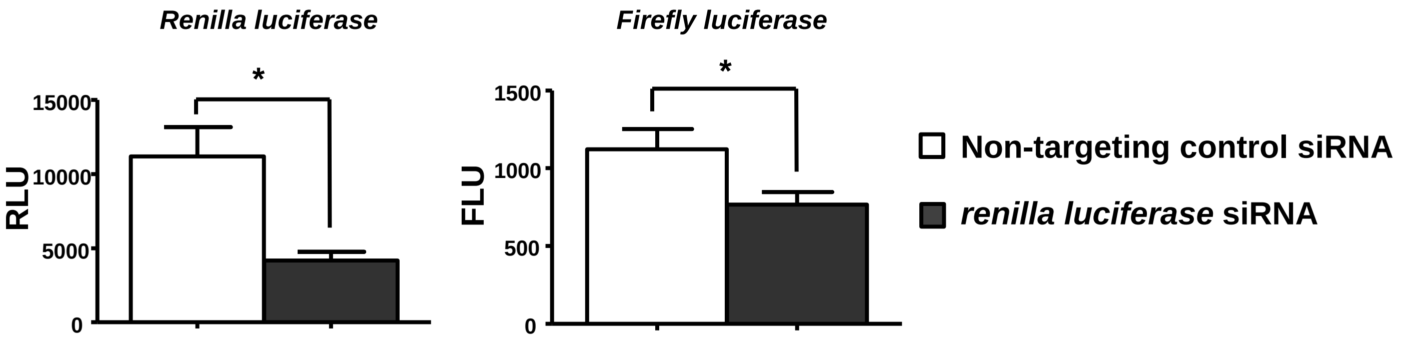

phpR-243-F

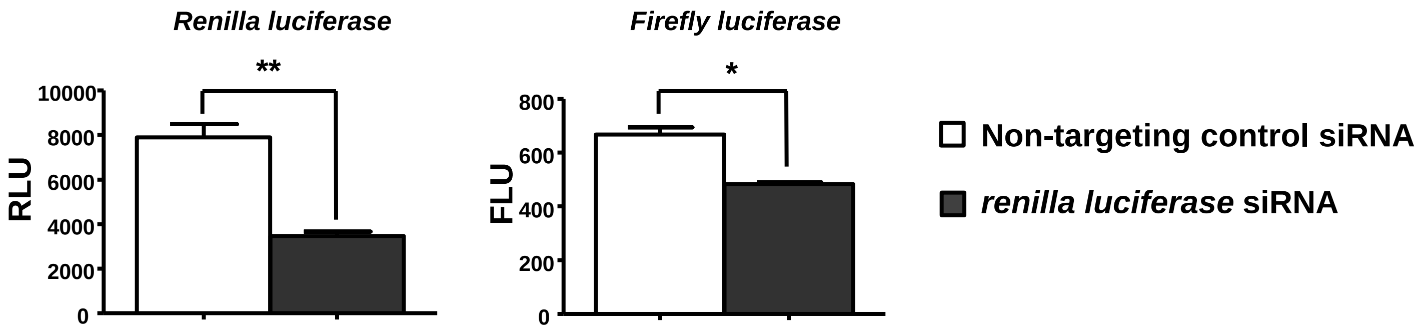

phpR-257-F

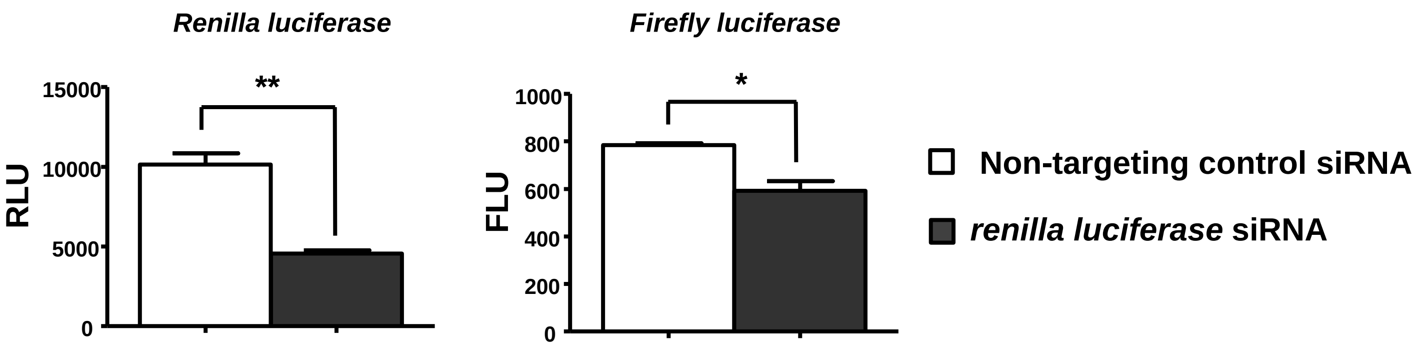

Supplementary Figure S9

(D)

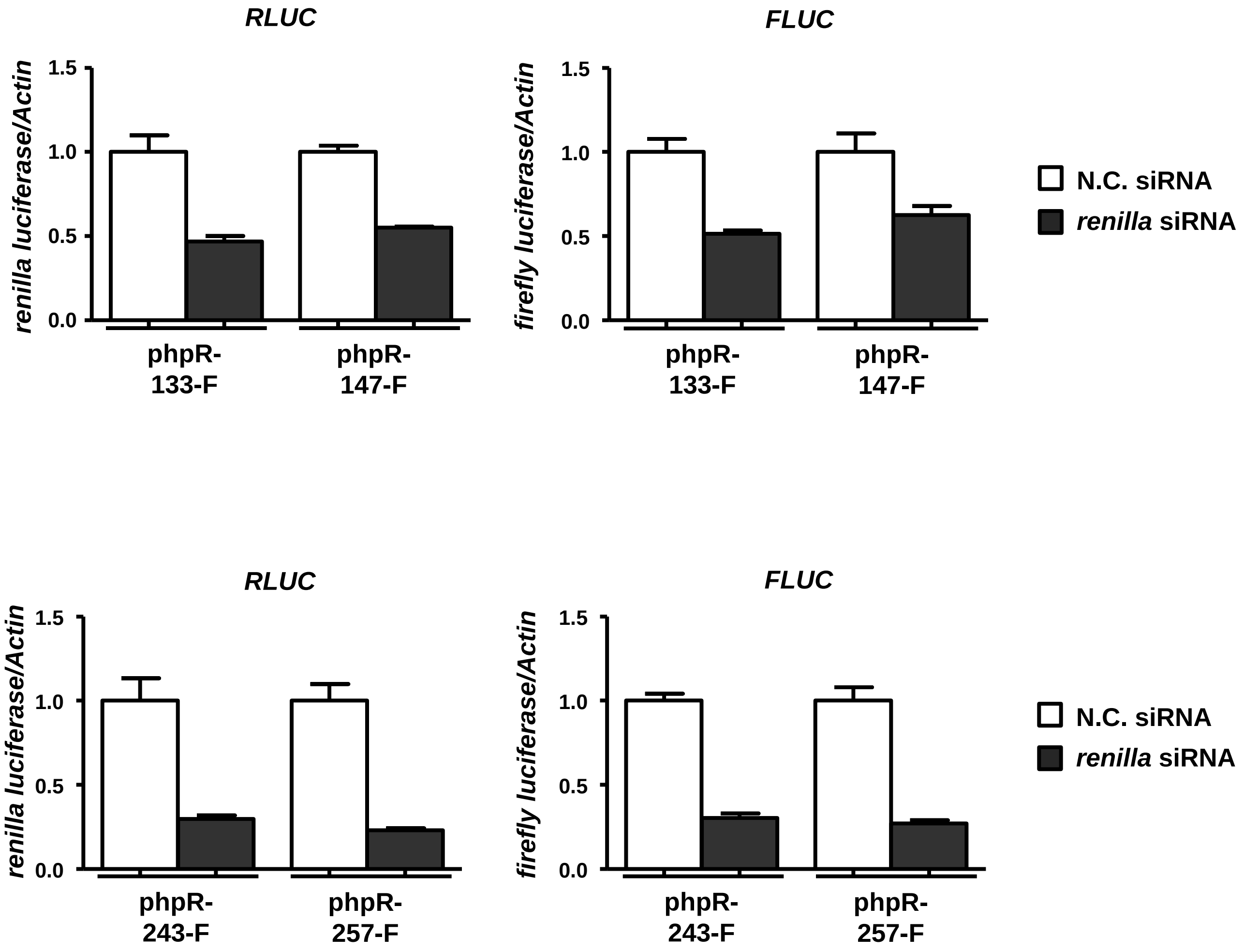

Supplementary Figure S10

(A)

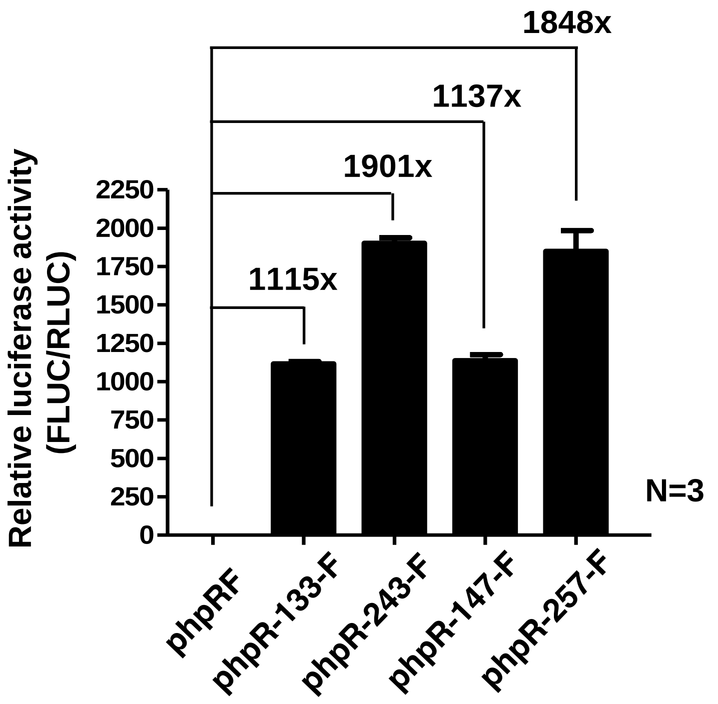

(B)

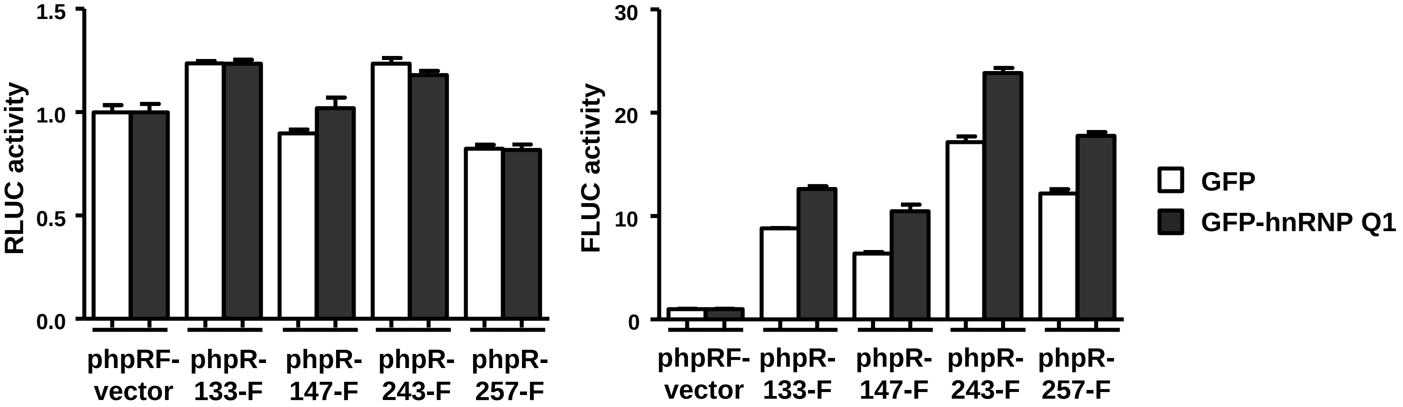

Supplementary Figure S10

(C)

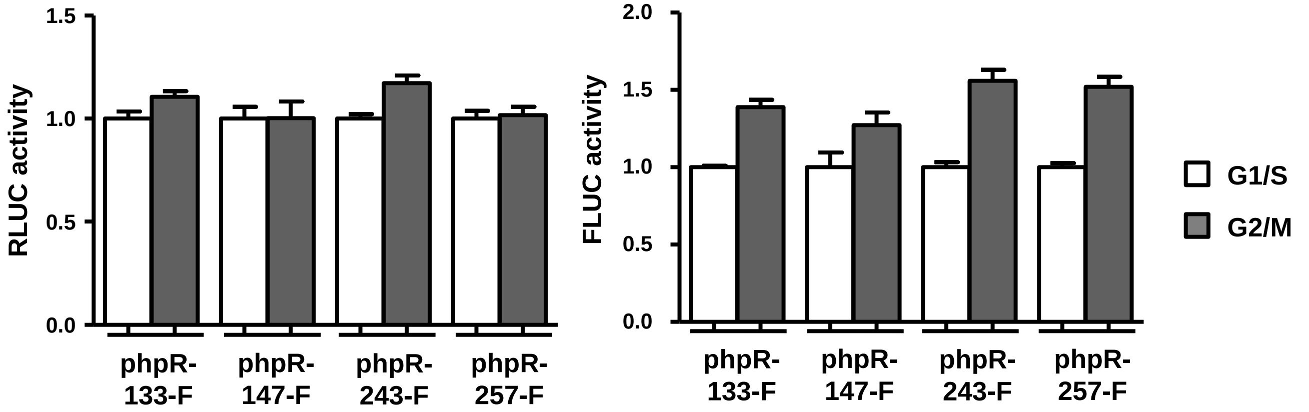

(D)

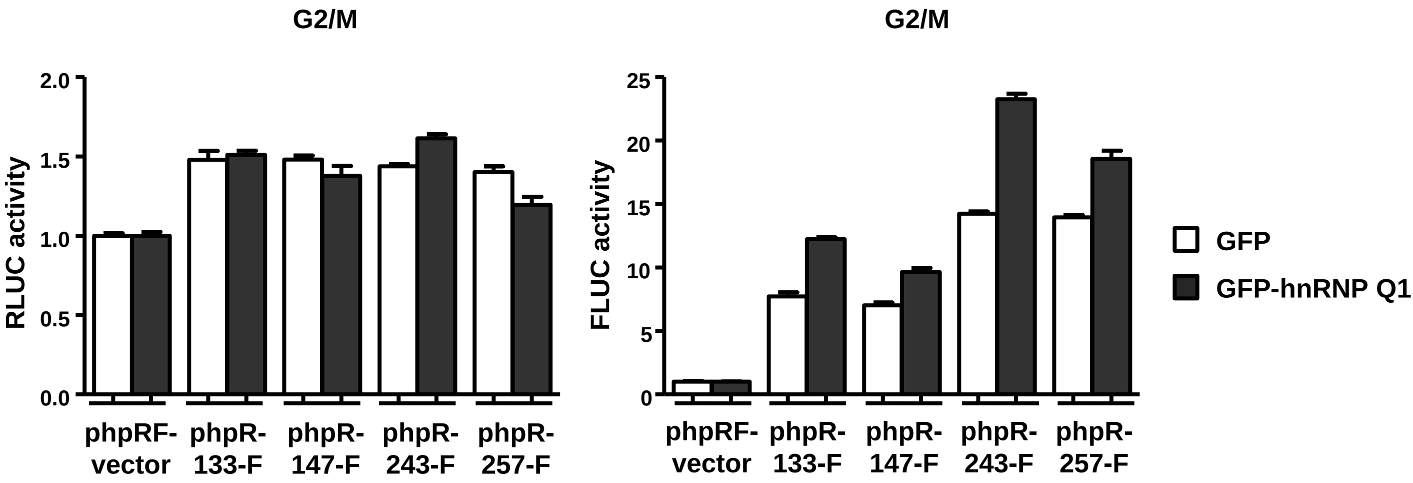

(E)

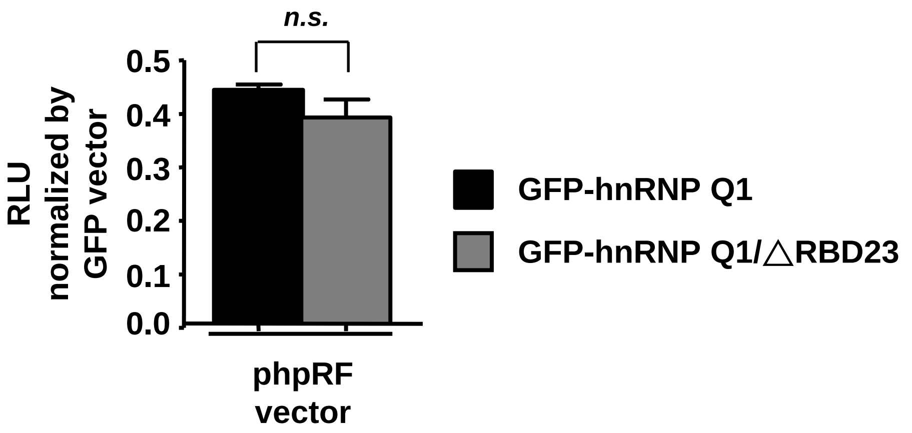

Supplementary Figure S11

(A)

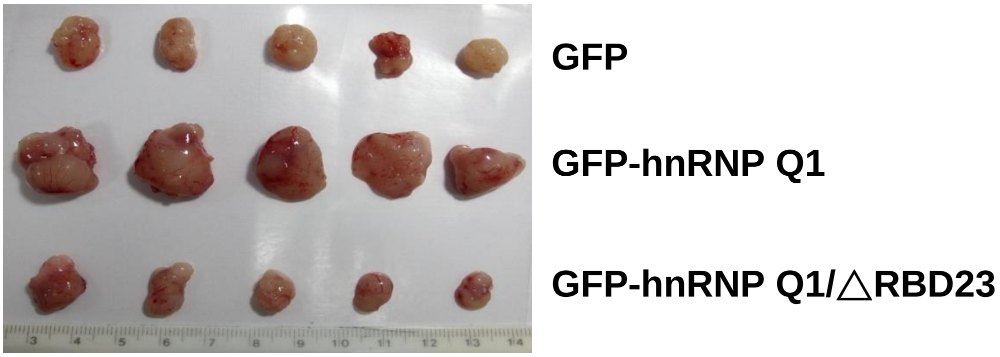

(B)

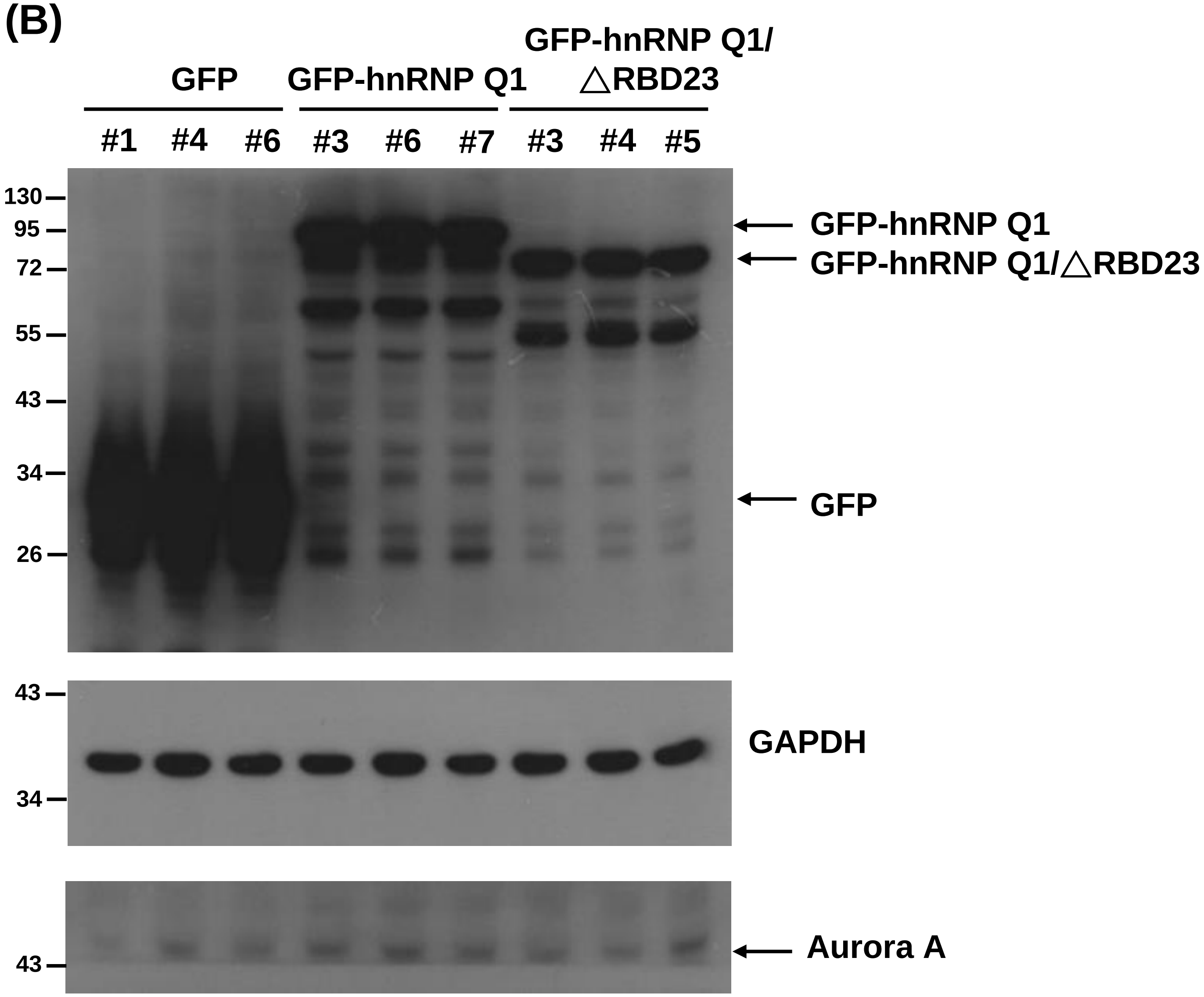

Supplement: Supplementary Figures [file cddis2016479x1.pdf]
