# Supplementary material for: Translational upregulation of Aurora-A by hnRNP Q1 contributes to cell proliferation and tumorigenesis in colorectal cancer
Source: Cell Death Dis. 2017 Jan 12;8(1):e2555–. doi: 10.1038/cddis.2016.479 (PMC5386382; doi:10.1038/cddis.2016.479)
Supplement: Supplementary Figure Legends [file cddis2016479x2.docx]

**Supplementary figure legends**

**Supplementary Figure S1. (A) Stable expression of GFP, GFP-hnRNP Q1, and GFP-hnRNP Q1/ΔRBD23 in SW480 cells.** Total cell lysates from three stable cell lines SW480-GFP, SW480-GFP-hnRNP Q1, or SW48-GFP-hnRNP Q1/ΔRBD23 were harvested for Western blot analysis using anti-GFP antibody. **(B)** **Subcellular localization of endogenous hnRNP Q1.** The nuclear fraction and cytosolic fraction of SW480 cells were purified, and the expression of hnRNP Q1, hnRNP Q2/Q3, and hnRNP R was determined by Western blot analysis using anti-hnRNP Q antibody. Lamin A/C is a nuclear protein, and α-tubulin is a cytoplasmic marker.

**Supplementary Figure S2. The expression pattern of *Aurora-A* 5’-UTR isoforms in colorectal cancer cell lines. (A)** Schematic illustration of *Aurora-A* mRNA 5’-UTR isoforms. Arrows (red) indicate the primer pairs used for identifying the expression pattern of the *Aurora-A* 5’-UTR in cancer cells. **(B)** Total RNA from colorectal cancer cell lines was purified to perform RT-PCR. All colorectal cancer cells contained the same expression pattern of *Aurora-A* 5’-UTR isoforms, including 133, 147, 243, and 257 nt. **(C)** The *Aurora-A* 5’-UTR isoforms in SW480 and CRL1790 cells were analyzed by RT-qPCR. The levels of *Aurora-A*, *exon1-* and *exon2*-containing 5’-UTR were determined respectively and normalized by *Actin*. **(D)** Schematic illustration of hnRNP Q1 and truncated GST-hnRNP Q1 fragments. The three RNA binding domains (RBD1-3) and one RGG domain of hnRNP Q1 are shown. Truncated GST-hnRNP Q1 recombinant proteins were generated and analyzed by Coomassie blue staining. Bovine serum albumin (BSA) was a quantitative control.

**Supplementary Figure S3. The subcellular localization of GFP-hnRNP Q1 and GFP-hnRNP Q1/ΔRBD23.** Immunofluorescence assay showed the subcellular localization of GFP-hnRNP Q1 or GFP-hnRNP Q1/ΔRBD23 (green) in SW480 stable cell lines. DAPI is a DNA-specific dye.

**Supplementary Figure S4. (A)** The schematic illustration of *Aurora-A* mRNA 5’-UTR 147 nt (147-F) and the fragments 147-A, 147-B and 147-C used in i*n vitro* biotin pull-down assay. **(B)** The secondary structure of *Aurora-A* 5’-UTR 147 nt predicted by mFold. **(C)** Biotin-labeled *Aurora-A* mRNA 5’-UTR 147 nt RNA probe (F) or the fragments (A, B and C) were incubated with cell lysates from GFP-hnRNP Q1-expressing cells to perform i*n vitro* biotin pull-down assay. The pulled-down proteins were separated by SDS-PAGE and immunoblotted with anti-GFP antibody.

**Supplementary Figure S5. (A)** **The expression level of hnRNP Q1 did not interfere with the protein stability of Aurora-A.** SW480 cells were transfected with control siRNA (left) or hnRNP Q siRNA (right) and then treated with cycloheximide (200 μg/ml) for various time periods as indicated. The expression of Aurora-A or hnRNP Q1 was determined by Western blot analysis. The expression levels of Aurora-A protein were measured and normalized to α-tubulin. Three different independent experiments were performed and the quantitative results are shown below. **(B)** **HnRNP Q1 did not interfere with the expression level of *Aurora-A* mRNA.** SW480 cells transiently transfected with GFP, GFP-hnRNP Q1, control siRNA (NC), or hnRNP Q siRNA were harvested to detect the expression level of *Aurora-A* mRNA by RT-qPCR from three independent experiments. **(C)** **HnRNP Q1 did not alter the expression level of *Aurora-A* mRNA 5’-UTR.** SW480 cells transiently transfected with GFP, GFP-hnRNP Q1, control siRNA (NC), or hnRNP Q siRNA were harvested to detect the expression of *Aurora-A* mRNA 5’-UTR by RT-PCR.

**Supplementary Figure S6.** **(A) GFP-hnRNP Q1, but not GFP-hnRNP Q1/ΔRBD23, can increase the expression of Aurora-A in HCT116 cells.** HCT116 cells with GFP vector, GFP-hnRNP Q1, or GFP-hnRNP Q1/ΔRBD23 expression were collected to determine the expression level of Aurora-A by Western blot analysis. **(B) HnRNP Q1 enhances *Aurora-A* 5’-UTRs’ translational activity.** SW480 cells were co-transfected with GFP or GFP-hnRNP Q1 and four types of *Aurora-A* 5’-UTR isoforms containing reporter (133-pGL3, 147-pGL3, 243-pGL3, and 257-pGL3), and then the *in vivo* translation assay was performed. The expression levels of *luciferase* mRNAs and GFP or GFP-hnRNP Q1 proteins were detected by RT-qPCR and Western blot, respectively. RLU, relative luciferase unit. **(C)** **GFP-hnRNP Q1 has no effect neither on pGL3-promoter vector nor *WWOX*-5’UTR-pGL3.** SW480 cells were co-transfected pGL3 plasmids and GFP-hnRNP Q1 for 24 hours and performed in vivo translation assay. Neither pGL3-promoter vector nor *WWOX*-5’UTR-pGL3 luciferase level was altered by GFP-hnRNP Q1. **(D) HnRNP Q1/ΔRBD23 did not increase the translational activity of *Aurora-A* 5’-UTR as hnRNP Q1.** Cells with GFP, GFP-hnRNP Q1, or GFP-hnRNP Q1/ΔRBD23 expression were used to perform the *in vivo* translation assay using *Aurora-A* mRNA 5’-UTR 147 nt reporter (147-pGL3). **(E) eIF-4E binding on *Aurora-A* mRNA is increased in hnRNP Q1 stably expressing cell.** RNA immunoprecipitation by eIF-4E was performed in GFP or GFP-hnRNP Q1 stably expressing SW480 cells, and the amount of eIF-4E associating RNAs was analyzed by RT-PCR (upper) and RT-qPCR (lower). *γ-actin* was used as negative control. **, p* < 0.05.

**Supplementary Figure S7. Exogenous expressed Aurora-A in hnRNP Q1/ΔRBD23 stable cells restores the proliferation enhancement effect as hnRNP Q1 stable cells. (A)** Proliferation assay was performed by CCK-8 kit and the proliferation rate was evaluated by O.D. 450 nm absorbance at day 1 and day 4. **(B)** For colony formation assay, each 3.5 mm dish was seeded with 1000 cells and harvested after 12 days. The colonies were stained with crystal violet and quantified by Image J.

**Supplementary Figure S8. (A) The expression level of hnRNP Q1 does not change during cell cycle progression.** GFP (left) or GFP-hnRNP-Q1 (right) stable cells were synchronized by double thymidine treatment and then released for different time periods. Total cell lysates were harvested at different time points for Western blot analysis using antibodies as indicated. **(B)** **The overexpression of GFP-hnRNP Q1 increases the level of phospho-histone H3/serine10.** Total cell lysates from GFP, GFP-hnRNP Q1, or GFP-hnRNP Q1/ΔRBD23-expressing cells were collected to perform Western blot analysis using antibodies as indicated.

**Supplementary Figure S9. (A) (Upper)** Schematic illustration of the bicistronic reporter constructs, pRF vector and phpRF vector, of the four *Aurora-A* 5’-UTR isoforms. *RLUC*, *renilla luciferase*; *FLUC*, *firefly luciferase*. **(Lower)** A representative example examines the IRES activity of *Aurora-A* 5’-UTR. SW480 cells were transfected with pR-133-F or phpR-133-F plasmid for 24 hours, and the translational activity was determined by *in vivo* translation assay. Cap-dependent translation is evaluated by renilla luciferase activity, and IRES translational activity is determined by firefly luciferase activity. ****, *p* < 0.01. **(B) Cryptic promoter or splicing is excluded in all phpR-*Aurora-A* 5’-UTRs-F plasmids.** Each phpRF plasmid was transfected into SW480 cells for 24 hours, and the total RNA was harvested for analyzing by RT-PCR using primer pair RLUC-F (5’-ATGATCCAGAACAAAGGAAACG-3’, at position: 17-38 of 936 bp) and FLUC-R (5’-GCCTTTATGAGGATCTCTCTGA-3’, at position: 1899-1920 of 1948 bp) as indicated. **(C) The confirmation of *Aurora-A* 5’-UTRs IRES activity in the bicistronic reporter phpRF vector.** SW480 cells were co-transfected with four phpR-*Aurora-A* 5’-UTRs-F plasmids and *renilla luciferase* siRNA (100 nM) for 24 hours and then performed the *in vivo* translation assay. The firefly luciferase activity was decreased when *renilla* *luciferase* gene was knocked down. ***, *p* < 0.05; ****, *p* < 0.01. **(D)** SW480 cells co-transfected with phpRF plasmids and *renilla luciferase* siRNA were harvested to evaluate the expression levels of *renilla* and *firefly luciferase* mRNA by RT-qPCR.

**Supplementary Figure S10. (A and B) *Aurora-A* 5’-UTRs contain IRES activity.** SW480 cells were transfected with different phpR-*Aurora-A* 5’-UTR-F plasmids (**A**), or co-transfected with GFP vector or GFP-hnRNP Q1 (**B**). The IRES activity was determined by *in vivo* translation assay. The activity of renilla luciferase (RLUC) and firefly luciferase (FLUC) were evaluated respectively. phpRF vector is a negative control. **(C)** SW480 cells transfected with different phpR-*Aurora-A* 5’-UTR-F plasmids were synchronized at G1/S phase or G2/M phase by 2 mM thymidine or 50 ng/ml nocodazole, and determined their IRES activity as described above. **(D)** SW480 cells were co-transfected with different phpR-*Aurora-A* 5’-UTR-F plasmids and GFP vector or GFP-hnRNP Q1, and then synchronized at G2/M phase by nocodazole. The IRES activity was determined as described above. **(E)** Cells with phpRF vector only were transfected with GFP-hnRNP Q1 or GFP-hnRNP Q1/ΔRBD23 and then determined their IRES activity. *n.s.*, non-significant.

**Supplementary Figure S11. (A)** Representative images of xenograft tumors from GFP, GFP-hnRNP Q1 or GFP-hnRNP Q1/ΔRBD23 stably expressed SW480 cells. (B) Total cell lysates from xenograft tumors were purified to check the expression of GFP, GFP-hnRNP Q1 or GFP-hnRNP Q1/ΔRBD23 by Western Blot analysis. Expression level of Aurora-A protein in these tumor samples is also shown. GAPDH is a loading control.
