# Supplementary material for: Translational upregulation of Aurora-A by hnRNP Q1 contributes to cell proliferation and tumorigenesis in colorectal cancer
Source: Cell Death Dis. 2017 Jan 12;8(1):e2555–. doi: 10.1038/cddis.2016.479 (PMC5386382; doi:10.1038/cddis.2016.479)
Supplement: Supplementary Information [file cddis2016479x3.docx]

**Supplementary Materials and Methods**

**Cell culture**

SW480 cells were cultured in Leibovitz’s L-15 medium (Gibco, Invitrogen, Carlsbad, CA); HCT116 cells were cultured in RPMI medium 1640 (Gibco), and CRL1790 cells were cultured in Minimum Essential Medium (MEM) with 1 mM Sodium Pyruvate (Gibco). All media were supplemented with 10% fetal bovine serum, 100 μg/ml streptomycin, and 100 unit/ml penicillin. The GFP, GFP-hnRNP Q1, and hnRNP Q1/△RBD23 stable clones were established in SW480 and selected by 2 mg/ml G418 (Gibco).

**Plasmid construction, siRNA and transfection**

For cap-dependent translational assay, *Aurora-A* 5’-UTR isoforms were subcloned into pGL3-promoter vector (Promega) as described previously ([1](#_ENREF_1)). For the bicistronic reporter system, *Aurora-A* 5’-UTRs were inserted into phpRF vector ([2](#_ENREF_2), [3](#_ENREF_3)). HnRNP Q1 cDNA was obtained from LS174T cells by RT-PCR using forward primer 1: 5’-GTCCGGAATGGCTACAGAACATGTTAATG-3’ and reverse primer 1: 5’-GTCGACTCATTGTAACAGGTCAGGACCG-3’, and subcloned into pEGFP-C1 vector (Clontech) by BspEI and SalI sites. HnRNP Q1/ΔRBD23 was prepared from hnRNP Q1 cDNA by ligating two cDNA fractions obtained from RT-PCR using forward primer 1 and reverse primer 2: 5’-CCTATTGTTGGCAACTGAGATGC-3’, and forward primer 2: 5’-GCCAAGCCACCAGATCAGAAAAG-3’ and reverse primer 1. The *hnRNP Q* (*SYNCRIP*) siRNA and *eIF-4E* siRNA were purchased from Dharmacon^®^. All plasmids or siRNAs were transfected into cells by Lipofectamine2000 (Invitrogen) according to the user instruction.

**Reverse-transcription polymerase chain reaction (RT-PCR) and real-time PCR**

The total RNAs were extracted by Trizol (Invitrogen) according to the manufacturer’s instruction, and reverse-transcripted to cDNA by Promega GoScript™ Reverse Transcription System. The primer sequences for PCR are described as follows: *GAPDH* forward, 5’-CCATCACCATCTTCCAGGAG-3’, reverse, 5’-CCTGCTTCACCACCTTCTTG-3’; *Aurora-A* forward, 5’-AATGCCCTGTCTTACTGTCATTC-3’, reverse, 5’-TCCAGAGATCCACCTTCTCATC-3’; *Aurora-A* 5’-UTR forward, 5’-GTCAACCAATCACAAGGCAGC-3’, reverse, 5’-CAGTTTTCTTTAGATCGGTCC-3’; *hnRNP Q1* forward, 5’-GTCGACGGAAAACATATTGGTGTCTG-3’, reverse, 5’-GCGGCCGCCCTTTTCTGATCTGGTGGC-3’. For real-time qPCR, the primer sequences are described as follows: *actin* forward, 5’-CTGGACTTCGAGCAAGAGATG-3’, reverse, 5’-TGATGGAGTTGAAGGTAGTTTCG-3’; *firefly luciferase* forward, 5’-GAGCACGGAAAGACGATG-3’, reverse, 5’-GCCTTTATGAGGATCTCTCTGA-3’; *renilla* *luciferase* forward, 5’-GGAGAATAACTTCTTCGTGGAAAC-3’, reverse, 5’-GCTGCAAATTCTTCTGGTTCTAA-3’; *γ-Actin* forward, 5’-CCCATCTACGAGGGCTACG-3’, reverse, 5’-TTCATGAGGTAGTCGGTCAGG-3’. *Aurora-A*, *Aurora-A 5’-UTR exon1*, and *exon2* primers using in real-time qPCR are the same as PCR as described above. The polymerase reagents used in PCR and Q-PCR are EconoTaq PLUS GREEN Master Mix Kit (Lucigen, F93481-1) and SYBR Advantage qPCR Premix (Clontech).

**Preparation of cell lysates and Western blot analysis**

For total cell lysates, cells were washed twice with PBS and lysed with RIPA buffer (50 mM Tris-HCl [pH 8], 150 mM NaCl, 0.1% NP-40, 0.5% sodium deoxycholate, 1 mM EDTA). 1 mM sodium orthovanadate, 1 mM phenylmethylsulfonyl fluoride, 1 mM DTT, 10 mg/ml leupeptin, and 10 mg/ml aprotinin were added into the RIPA buffer before use. For Western blot analysis, protein lysates were separated by SDS-PAGE and transferred to PVDF membrane by Turbo^TM^ Transfer Starter System (BioRad). After being blocked by 5% skim milk (in TBST buffer - 0.01 M Tris, 150 mM NaCl, 0.05% Tween-20) at room temperature for 1 h, the membrane was hybridized with primary antibodies at 4°C overnight, and then washed by TBST buffer and hybridized with secondary HRP-conjugated antibodies at room temperature for 1 h. The HRP signal was detected by Western Lightning® Plus-ECL (PerkinElmer). The antibodies used for protein detection are described below: anti-Aurora-A (35C1, GeneTex), anti-cyclin B1 (Santa Cruz), anti-p-4E-BP1/Thr70 (Cell Signaling), anti-4E-BP1 (Cell Signaling), anti-eIF-4E (P-2, Santa Cruz), anti-GAPDH (Santa Cruz), anti-p-histone H3 ser10 (Santa Cruz), anti-histone H3 (Santa Cruz), anti-α-tubulin (DM1A, Sigma), anti-Lamin A/C (636, Santa Cruz), anti-hnRNP Q (I8E4, Santa Cruz), anti-ribosomal protein S6 (C-8, Santa Cruz) and anti-GFP (JL-8, Clontech).

**Proliferation assay**

SW480 cells were seeded in 96-well plate by 1500 cells/well. Every 24 h, the cell number was measured by CCK-8 kit (Dojindo Molecular Technologies, Rockville, Maryland) according to the manufacturer’s instructions.

**Colony formation assay**

SW480 cells were seeded in 6-well plate by 2000 cells/well for around 3 weeks until the development of the colonies. The colonies were stained by crystal violet and calculated by Image J.

**Preparation for Ribo-seq and RNA-seq**

This protocol is adapted from the protocol of Dr. Ingolia with moderate modification ([4](#_ENREF_4)). Briefly, cells were lysed with polysome buffer (20 mM Tris-HCl, pH7.4; 150 mM NaCl; 5 mM MgCl2; 1 mM DTT; 200µg/ml cyclohexamide; 1 % Triton X-100 and 25U/ml Turbo DNase I (AM2238, Ambion)), and lysate were digested with RNAse I (AM2294, Ambion). The rRNA was depleted with Ribo-Zero™ rRNA removal kits (MRZH11126, Illumina) from the purified and enriched small RNA. For RNAseq library construction, total RNA was extracted and oligo-dT magnetic beads were used to select RNA containing poly-A tails. Selected RNA was fragmented and 50-60 nucleotides-long was selected following end-repairing, linker ligation, reverse transcription and circular PCR.

Ribosome-protected fragments (RPF) purified with PAGE/fragmented total RNA were dephosphorylated with T4-PNK (M0201S, NEB). Linkers were ligated to RPF/fragmented total RNA by T4 Rnl2tr K227Q ligase (M0351L, NEB) and the cDNA synthesis was performed with SuperScriptIII (18080-093, Invitrogen). The cDNA samples were circularized by CircLigase (CL4111, Epicentre). The DNA libraries were amplified with Phusion® High-Fidelity DNA Polymerase (M0530S, NEB) and purified with AMPure XP system (A63880, Beckmancoulter). The quantified libraries were sequenced with Illumina sequencing platform following the manufacturer’s instruction.

**Cell cycle synchronization**

Double thymidine treatment was used to obtain the synchronized G1/S phase cells. Briefly, the cells were treated with 2 mM thymidine for 18 h and replaced by fresh medium for 8-10 h, and then re-treated with 2 mM thymidine for 18 h. For G2/M phase synchronization, cells were treated with 45 ng/ml nocodazole for 16 h, and the round-up mitotic cells were collected by shake-off.

**Xenograft animal model**

Male **NOD-SCID** mice at an age of around 5~6 weeks were purchased from National Cheng Kung University Laboratory Animal Center and National Laboratory Animal Center, and all animal studies were according to protocols approved by Laboratory Animal Committee of National Cheng Kung University. Mice were injected with SW480 cells, which stably expressed GFP, GFP-hnRNP Q1, and hnRNP Q1/△RBD23. A total of 5X10^6^ cells were subcutaneously injected into the right flank of each mouse. The mice were monitored for the appearance of tumors and were sacrificed at the indicated times. The weight of the tumors was measured.

**Immunohistochemistry staining**

Immunohistochemistry staining was performed as described previously ([5](#_ENREF_5)). Antibodies used for IHC staining are anti-Aurora-A (ab13824/35C1, Abcam) and anti-hnRNP Q1 (AP7852c, Abgent).

**References**

1. Lai CH, Tseng JT, Lee YC, Chen YJ, Lee JC, Lin BW, et al. Translational up-regulation of Aurora-A in EGFR-overexpressed cancer. Journal of cellular and molecular medicine. [Research Support, Non-U.S. Gov't]. 2010 Jun;14(6B):1520-31.

2. Coldwell MJ, Mitchell SA, Stoneley M, MacFarlane M, Willis AE. Initiation of Apaf-1 translation by internal ribosome entry. Oncogene. 2000 Feb 17;19(7):899-905.

3. Stoneley M, Paulin FE, Le Quesne JP, Chappell SA, Willis AE. C-Myc 5' untranslated region contains an internal ribosome entry segment. Oncogene. 1998 Jan 22;16(3):423-8.

4. Ingolia NT, Brar GA, Rouskin S, McGeachy AM, Weissman JS. The ribosome profiling strategy for monitoring translation in vivo by deep sequencing of ribosome-protected mRNA fragments. Nat Protoc. 2012 Aug;7(8):1534-50.

5. Hughes S, Williams RD, Webb E, Houlston RS. Meta-analysis and pooled re-analysis of copy number changes in colorectal cancer detected by comparative genomic hybridization. Anticancer Res. 2006 Sep-Oct;26(5A):3439-44.
