# Supplementary material for: Translational upregulation of Aurora-A by hnRNP Q1 contributes to cell proliferation and tumorigenesis in colorectal cancer
Source: Cell Death Dis. 2017 Jan 12;8(1):e2555–. doi: 10.1038/cddis.2016.479 (PMC5386382; doi:10.1038/cddis.2016.479)
Supplement: Supplementary Tables [file cddis2016479x4.docx]

**Supplementary Table S1. Gene ontology of hnRNP Q1 targeting genes**

| **Gene ontology of hnRNP Q1 targeting genes from NGS data (Top 10 according to P-value)** | | | |
| --- | --- | --- | --- |
| Term | Gene | % | P-Value |
| GO:0006412~translation | 161 | 7.152376721 | 3.00E-56 |
| GO:0006414~translational elongation | 83 | 3.687250111 | 9.04E-55 |
| GO:0006396~RNA processing | 172 | 7.641048423 | 5.45E-30 |
| GO:0008380~RNA splicing | 109 | 4.842292315 | 6.02E-27 |
| GO:0006511~ubiquitin-dependent protein catabolic process | 97 | 4.309195913 | 1.08E-25 |
| GO:0016071~mRNA metabolic process | 126 | 5.597512217 | 1.74E-25 |
| GO:0006397~mRNA processing | 111 | 4.931141715 | 4.08E-23 |
| **GO:0000278~mitotic cell cycle** | 121 | 5.375388716 | 8.69E-23 |
| GO:0046907~intracellular transport | 178 | 7.907596624 | 1.00E-22 |
| **GO:0007049~cell cycle** | 197 | 8.751665926 | 1.60E-21 |

% indicates the percentage of total genes from RNA-IP-seq.

**Supplementary Table S2. HnRNP Q1 targeting genes involved in mitotic cell cycle**

| **HnRNP Q1 targeting genes involved in mitotic cell cycle (Total 121 genes)** |
| --- |
| *PRC1, DBF4, TTK, AURKA, CDC16, PTTG1, CUL3, APP, CUL5, RAD21, TARDBP, CDCA2, DNAJC2, CCNA2, CUL1, STAG2, RPS27A, STAG1, EGFR, RAN, PIM1, SKP1, PPP1CB, NCAPD2, DCTN2, PSMA1, MAD2L1, PSMA6, PSMA4, PSMA3, STMN1, CCDC99, ANLN, CHEK1, PSMA7, CCNG1, ITGB1, PSMB4, PSMB7, PSMB1, PSMB3, CDC123, PBRM1, SKA3, SKA2, HELLS, CKAP5, SUGT1, CDC26, CDC27, NEDD1, PSMC6, PSMC5, PLK2, NOLC1, PSMC3, PSMC2, RNF2, PSMC1, UBE2E1, E2F6, NDE1, SEH1L, PSMD1, PSMD2, PSMD5, PSMD6, USP16, PSMD7, CDC6, CDK1, CCNK, NUSAP1, UBE2I, CDK6, RB1, HMGA2, EML4, RBBP8, CCND1, UBC, CDK2AP1, BUB1B, MDM2, PSME3, UBB, MAPRE1, KPNA2, GADD45A, HAUS3, USP9X, HAUS2, CEP55, NCAPG, BUB1, PAFAH1B1, ZWILCH, BOD1, PDS5B, PDS5A, CENPF, RPL24, CENPE, CDKN3, CEP63, SMC3, MIS12, CDC25B, SMC4, CCNB1, MPHOSPH9, PSMD14, CCNB2, PSMD13, PSMD12, CUL4A, GSPT1, PSMD11, SETD8, MPHOSPH6, TXNL4A* |
